# Benzaldehyde suppresses epithelial-mesenchymal plasticity and overcomes treatment resistance in cancer by targeting the interaction of 14-3-3ζ with H3S28ph

**DOI:** 10.1038/s41416-025-03006-4

**Published:** 2025-05-02

**Authors:** Jun Saito, Nobuyuki Onishi, Juntaro Yamasaki, Naoyoshi Koike, Yukie Hata, Kiyomi Kimura, Yuji Otsuki, Hiroyuki Nobusue, Oltea Sampetrean, Takatsune Shimizu, Shogo Okazaki, Eiji Sugihara, Hideyuki Saya

**Affiliations:** 1 Oncology Innovation Center, Fujita Health University, Toyoake, Aichi 470-1192 Japan; 2 Department of Pathophysiology, Hoshi University, Shinagawa, Tokyo 142-0063 Japan; 3 Department of Microbiology and Immunology, Nihon University School of Dentistry, Chiyoda, Tokyo 101-0062 Japan; 4 Department of Applied Physics and Chemistry, The University of Electro-Communications, Chofu, Tokyo 182-8585 Japan; 5 Ichijokai Hospital, Ichikawa, Chiba, 272-0836 Japan; 6 Department of Clinical Diagnostics Oncology, Clinical Research Institute for Clinical Pharmacology and Therapy, Showa University, Shinagawa, Tokyo 142-8555 Japan; 7 Department of Plastic and Reconstructive Surgery, Keio University School of Medicine, Shinjuku, 160-8582 Tokyo Japan; 8 Department of Radiology, Keio University School of Medicine, Shinjuku, Tokyo 160-8582 Japan; 9 Department of Breast Oncology Juntendo University School of Medicine, Bunkyo, Tokyo 113-0033 Japan; 10 Keio University Human Biology-Microbiome-Quantum Research Center (WPI-Bio2Q), Shinjuku, Tokyo 160-8582 Japan

**Keywords:** Cancer stem cells, Cancer therapy

## Abstract

**Background:**

Benzaldehyde (BA) is an aromatic aldehyde found in fruits that has been studied as a potential anticancer agent on the basis of its ability to inhibit transformation in mouse embryo cells and to suppress metastasis in mice.

**Methods:**

We investigated the cytotoxic effects of BA on cancer cells, and probed its effects on intracellular signaling pathways. The anticancer effects of BA in vivo were studied by using a mouse orthotopic transplantation model of pancreatic cancer.

**Results:**

BA inhibited the growth of osimertinib- or radiation-resistant cancer cells as well as the interaction between 14-3-3ζ and its client proteins. The interaction of 14-3-3ζ with the Ser^28^-phosphorylated form of histone H3 (H3S28ph) was implicated in treatment resistance and the transcriptional regulation of genes related to epithelial-mesenchymal transition and stemness, including *E2F2*, *SRSF1*, and *ID1*. Treatment of mice with a BA derivative inhibited pancreatic tumor growth and lung metastasis, as well as suppressed a state of epithelial-mesenchymal plasticity (EMP) of tumor cells.

**Conclusion:**

The interaction between 14-3-3ζ and H3S28ph plays a key role in EMP and treatment resistance in cancer. The ability of BA to inhibit this and other interactions of 14-3-3ζ offers the potential to overcome treatment resistance and to suppress metastasis.

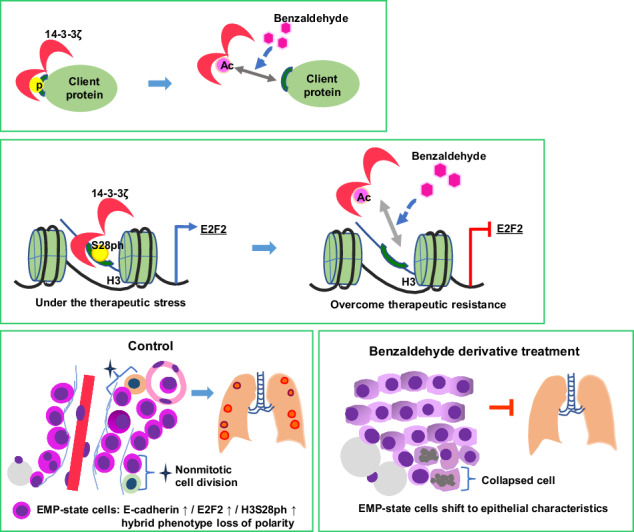

## Introduction

Benzaldehyde (BA) is the simplest aromatic aldehyde and is present in various fruits including almonds, apricots, and figs. In the 1980s, several BA derivatives were developed in Japan and showed potential efficacy in clinical trials for lung cancer [1]. Although these derivatives showed minimal harm to normal cells and had a favorable safety profile in patients, their clinical effectiveness was not evaluated according to the efficacy criteria of the time, and their development was halted as a result of societal factors. Foundational data from that period revealed an inhibitory effect of BA on the spontaneous transformation of C3H/He mouse embryo cells [2], suppression of mouse lung metastasis by BA derivatives [3], and inhibition by BA of the proliferation of SV40-transformed cells but not of noncancerous parental cells [4, 5]. These findings suggested the potential of BA to prevent metastasis and to overcome treatment resistance, both of which are major challenges in cancer therapy.

The 14-3-3 family consists of acidic proteins that range in size from 28 to 30 kDa, bind to phosphorylated motifs, and are widely expressed in eukaryotes. Seven isoforms of 14-3-3 proteins (β, σ, γ, ε, η, θ, and ζ) have been identified in humans. Members of the 14-3-3 family maintain the phosphorylated state of client proteins and thereby regulate various cellular functions including metabolism, the cell cycle, apoptosis, and signal transduction. Among these proteins, 14-3-3ζ is highly expressed in many human cancer types and is associated with a poor prognosis. It promotes the activity of signaling pathways related to carcinogenesis, metastasis, radioresistance, and chemoresistance, including those mediated by phosphatidylinositol 3-kinase (PI3K)–AKT, insulin-like growth factor (IGF)–insulin receptor (IR), extracellular signal–regulated kinase (ERK), and transforming growth factor–β [6–8]. Serine residues at positions 10 and 28 of histone H3 undergo phosphorylation (phosphorylated forms denoted as H3S10ph and H3S28ph) and possess the ARKS sequence motif, a client protein motif for 14-3-3ζ [9, 10]. Phosphorylation of H3 during mitosis is associated with chromatin condensation mediated by the kinase Aurora B, whereas stimulus-induced phosphorylation of a small fraction of H3 by mitogen- and stress-activated kinase (MSK) during interphase affects gene regulation and transcription activity [11, 12]. H3 phosphorylation has also been implicated in malignant transformation and carcinogenesis [13, 14], although mechanistic details remain unclear.

Epithelial-mesenchymal transition (EMT) is associated with various other cellular programs and functions, including cancer cell stemness, resistance to apoptosis, genomic instability, cancer drug resistance, and metabolic adaptation [15, 16]. It is also thought to be responsible for the loss of intercellular adhesion among epithelial cancer cells and the associated acquisition of migratory and invasive potential that allows the cells to become circulating tumor cells and thereby to contribute to cancer metastasis. However, cancer cells undergoing the metastatic process have been found to manifest an intermediate phenotype characterized by the coexistence of both epithelial and mesenchymal traits due to cellular plasticity [17, 18], a phenomenon referred to as epithelial-mesenchymal plasticity (EMP) [16]. DNA methylation and histone H3 modifications have both been implicated in the regulation of such plasticity [19, 20].

In the process of characterizing the tumor-suppressive action of BA, we identified an epigenetic mechanism mediated by inhibition of the interaction between 14-3-3ζ and H3S28ph that results in suppression of treatment resistance in cancer cells. Our findings suggest that the ability of BA to inhibit metastasis is related to suppression of EMP and that H3S28ph is a histone modification factor that regulates EMP.

## Results

### Osimertinib and radiation resistance are associated with increased BA sensitivity in cancer cells

**Fig. 1:**
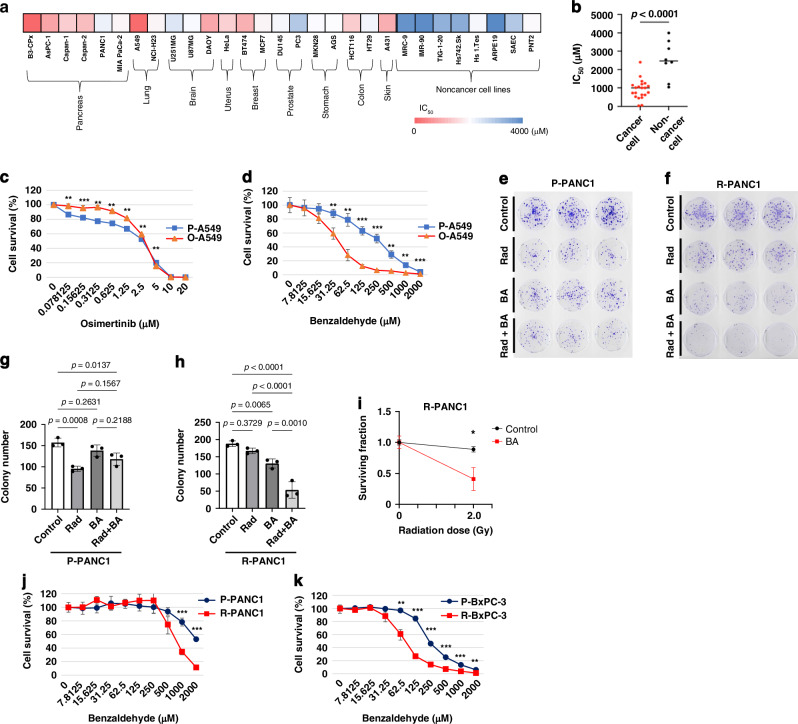
Osimertinib-resistant and radiation-resistant cancer cells show increased sensitivity to BA treatment. **a** Comparison of IC_50_ values for BA among 21 cancer cell lines derived from the indicated organs and 8 noncancer cell lines as determined by the XTT assay. Data are means from *n* = 2 independent experiments. **b** IC_50_ values for BA as in **a** showing the overall median values for cancer and noncancer cell lines. The *p* value was determined by the two-tailed Student’s *t* test. CellTiter-Glo assay for parental (P-A549) and osimertinib-resistant (O-A549) A549 cells exposed to the indicated concentrations of osimertinib (**c**) or BA (**d**) for 48 h. Data are means ± SD from *n* = 3 independent experiments. **p < 0.01, ***p < 0.001 versus the corresponding value for P-A549 (**c**) or O-A549 (**d**) cells (two-tailed Student’s *t* test). **e,** **f** Colony formation assay for parental (P-PANC1) and radiation-resistant (R-PANC1) PANC1 cells, respectively, exposed to 2 Gy of radiation (Rad) or to BA (1200 µM) and cultured for 14 days, as indicated. **g,** **h** Colony number determined as in **e** and **f**, respectively. Data are means ± SD from three independent experiments, and the *p* values were determined by one-way ANOVA followed by Tukey’s multiple-comparison test. **i** Surviving fraction for **f**, respectively. Data are means ± SD. *p < 0.05 versus BA (two-tailed Student’s *t* test). **j,** **k** CellTiter-Glo assay for P-PANC1 and R-PANC1 cells (**j**) as well as for parental (P-BxPC-3) and radiation-resistant (R-BxPC-3) BxPC-3 cells exposed to the indicated concentrations of BA for 48 h. Data are means ± SD from *n* = 3 independent experiments. **p < 0.01, ***p < 0.001 (two-tailed Student’s *t* test).

We first determined the median inhibitory concentration (IC_50_) of BA for 21 human cancer cell lines and 8 human noncancer cell lines with the use of the XTT assay of cell viability. The cytotoxicity of BA varied among the cell lines (Fig. 1a), with the IC_50_ for noncancer cell lines being significantly higher than that for cancer cell lines (Fig. 1b). Among the noncancer cell lines, PNT2, a prostate epithelial cell line immortalized by SV40, showed the lowest IC_50_, consistent with the previous finding that BA sensitivity is increased by SV40 infection [4, 5]. We next focused on the highly sensitive pancreatic cancer cell line BxPC-3 and lung cancer cell line A549 as well as the low-sensitivity pancreatic cell line PANC1.

To examine potential effects of cancer treatment resistance on BA sensitivity, we established A549 cells resistant to osimertinib, a tyrosine kinase inhibitor (TKI) for the epidermal growth factor receptor (EGFR) administered for the treatment of NSCLC. The osimertinib-resistant (O-A549) cells were generated by continuous exposure of parental A549 (P-A549) cells to 3 µM osimertinib for 10 days. A cell proliferation assay revealed that, whereas osimertinib sensitivity was significantly decreased in O-A549 cells relative to P-A549 cells (Fig. 1c), BA sensitivity was significantly increased (Fig. 1d), indicating that BA can mitigate osimertinib resistance.

We also investigated the potential of BA to overcome radiation resistance in PANC1 cells. We established radioresistant PANC1 (R-PANC1) cells by irradiating parental PANC1 (P-PANC1) cells with a total of 50 Gy (2 Gy/day, 5 days/week), and we then performed a colony formation assay with P-PANC1 (Fig. 1e) and R-PANC1 (Fig. 1f) cells exposed to BA at 1200 µM (similar to the IC_50_ for P-PANC1 cells) or to 2 Gy of radiation and incubated for 14 days. P-PANC1 cells showed a significant reduction in colony number after irradiation, whereas R-PANC1 cells did not (Fig. 1g, h). Conversely, BA treatment did not significantly affect colony number for P-PANC1 cells but significantly reduced that for R-PANC1 cells. The surviving fraction for R-PANC1 cells exposed to 2 Gy of radiation was significantly reduced by concomitant BA treatment (Fig. 1i), indicative of a synergistic effect of BA and radiation. A cell proliferation assay also showed that BA sensitivity was significantly increased in R-PANC1 cells compared with P-PANC1 cells (Fig. 1j).

In addition, we established radioresistant BxPC-3 (R-BxPC-3) cells by exposing parental (P-BxPC-3) cells to a total of 50 Gy of radiation, and we found that sensitivity to the growth-inhibitory effect of BA was significantly increased in the radiation-resistant cells compared with the parental cells (Fig. 1k). Together, these findings with PANC1 cells (low intrinsic sensitivity to BA) and BxPC-3 cells (high intrinsic sensitivity to BA) indicated that radioresistant cells show an increased sensitivity to BA, suggesting the potential of BA to overcome radiation resistance.

### BA inhibits multiple pathways upregulated in cancer by suppressing the interaction of 14-3-3ζ with client proteins

**Fig. 2:**
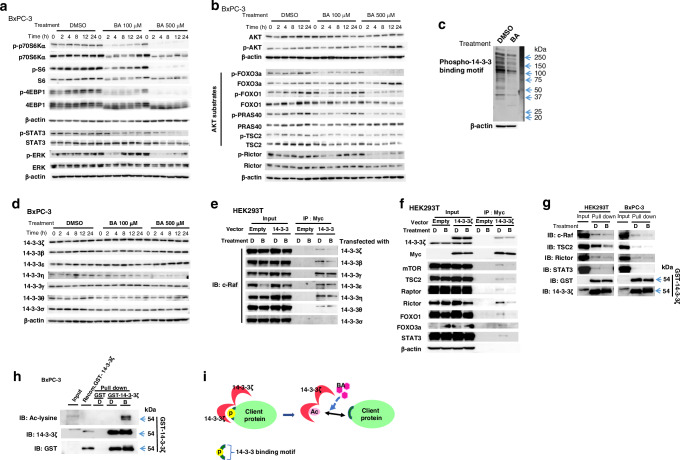
BA suppresses the activity of signaling pathways and inhibits interaction of 14-3-3ζ and its client proteins in cancer cells. **a** Immunoblot analysis of total and phosphorylated (p-) forms of proteins involved in cancer-associated signaling pathways in BxPC-3 cells incubated with BA (100 or 500 µM) or dimethyl sulfoxide (DMSO) vehicle for the indicated times. β-actin was examined as a loading control. **b** Immunoblot analysis of total and phosphorylated forms of AKT, of AKT substrates (FOXO3a, FOXO1, PRAS40, and TSC2), and of Rictor, which contributes to AKT phosphorylation, in BxPC-3 cells treated with BA as in **a**. **c** Immunoblot analysis of the phosphorylated 14-3-3 binding motif in BxPC-3 cells treated with DMSO or 500 µM BA for 24 h. **d** Immunoblot analysis of the seven isoforms of 14-3-3 proteins in BxPC-3 cells treated with BA as in **a**. **e** HEK293T cells transiently transfected with expression vectors for Myc epitope–tagged 14-3-3 protein isoforms (or with the empty vector) were incubated with DMSO (D) or 500 µM BA (B) for 24 h and then lysed and subjected to immunoprecipitation (IP) with antibodies to Myc. The resulting precipitates as well as the original cell lysates (Input) were subjected to immunoblot (IB) analysis with antibodies to c-Raf. **f** HEK293T cells transiently transfected with an expression vector for Myc epitope–tagged 14-3-3ζ (or with the empty vector) were incubated with DMSO (D) or 500 µM BA (B) for 24 h and then lysed and subjected to immunoprecipitation with antibodies to Myc. The resulting precipitates as well as the original cell lysates (Input) were subjected to immunoblot analysis with antibodies to mTOR, TSC2, Raptor, Rictor, FOXO1, FOXO3a, or STAT3. **g** HEK293T or BxPC-3 cells were treated with DMSO (D) or 1000 µM BA (B) for 100 min, lysed, and subjected to a pull-down assay with GST-tagged 14-3-3ζ. The resulting precipitates as well as the original cell lysates (Input) were subjected to immunoblot analysis with antibodies to c-Raf, TSC2, Rictor, STAT3, GST, or 14-3-3ζ. **h** BxPC-3 cells were treated with DMSO (D) or 1000 µM BA (B) for 100 min, lysed, and subjected to a pull-down assay with GST or GST-tagged 14-3-3ζ. The resulting precipitates as well as the original cell lysates (Input) were subjected to immunoblot analysis with antibodies to acetyl-lysine, 14-3-3ζ, or GST. The lane labeled Recom. GST–14-3-3ζ indicates recombinant GST-14-3-3 ζ without lysate. **i** Schematic illustration of the proposed mechanism by which BA induces acetylation of 14-3-3 ζ, its dissociation from client proteins, and consequent dephosphorylation of the client proteins.

To investigate the mechanism underlying the increased sensitivity of treatment-resistant cancer cells to BA, we examined the phosphorylation of proteins related to signaling pathways that are upregulated in association with cancer. Immunoblot analysis of BxPC-3 cells (highly sensitive to BA) revealed that BA treatment inhibited the phosphorylation of downstream targets of mechanistic target of rapamycin (mTOR)—including p70 S6 kinase (p70S6K), ribosomal protein S6, and the translational repressor 4EBP1—as well as that of ERK and signal transducer and activator of transcription 3 (STAT3) in a concentration- and time-dependent manner (Fig. 2a). In addition, BA treatment suppressed the phosphorylation of the AKT substrates FOXO1, FOXO3a, PRAS40, and TSC2 [21] (Fig. 2b). Whereas BA also inhibited the phosphorylation of Rictor, which contributes to AKT phosphorylation [21], the phosphorylation of AKT itself was not substantially affected (Fig. 2b), suggesting that the BA-mediated suppression of the phosphorylation of FOXO1, FOXO3a, PRAS40, and TSC2 was independent of AKT. Similar inhibition of protein phosphorylation related to multiple signaling pathways was also observed in A549 cells (Fig. S1a, S1b).

Members of the 14-3-3 family regulate intracellular signaling by binding to phosphorylated sites on various proteins and maintaining their phosphorylation state, and thereby play a key role in cell processes such as proliferation, differentiation, and apoptosis. Many such 14-3-3 client proteins have been identified in multiple pathways that are activated in cancer [6, 8]. We therefore hypothesized that the inhibition of signaling pathways by BA might be mediated through 14-3-3 proteins. Immunoblot analysis with antibodies to the phosphorylated 14-3-3 binding motif of client proteins revealed a marked decrease in the intensity of the blot signals for BxPC-3 cells after treatment with 500 µM BA for 24 h (Fig. 2c). Examination of the expression of the seven isoforms of 14-3-3 proteins (β, ε, γ, η, θ, σ, and ζ) in BxPC-3 cells showed that BA treatment substantially reduced the abundance of only 14-3-3η (Fig. 2d). Our results therefore suggested that BA inhibits the interaction between 14-3-3 and client proteins and thereby downregulate phosphorylation of the latter proteins and disrupt intracellular signaling.

Immunoprecipitation of the seven Myc epitope–tagged 14-3-3 isoforms expressed in transiently transfected HEK293T cells revealed that the interaction between the 14-3-3 client protein c-Raf and each of 14-3-3β and 14-3-3ζ was attenuated by treatment of the cells with 500 µM BA for 24 h (Fig. 2e). Focusing on 14-3-3ζ which was reported to be highly expressed in various cancers [6–8], we found that BA also inhibited the binding of 14-3-3ζ to TSC2, Rictor, Raptor, mTOR, FOXO1, FOXO3a, and STAT3 (Fig. 2f).

We also conducted pull-down assays with recombinant glutathione S-transferase (GST)–tagged 14-3-3ζ and lysates of HEK293T or BxPC-3 cells. BA inhibited the interaction of GST–14-3-3ζ with client proteins including c-Raf, TSC2, Rictor and STAT3 in both cell lines (Fig. 2g). Together, our results thus indicated that BA inhibits protein-protein interaction between 14-3-3ζ and multiple client proteins. Moreover, the pull-down assay with BxPC-3 cell lysates followed by immunoblot analysis with antibodies to acetyl-lysine revealed that GST-tagged 14-3-3ζ became acetylated on incubation with the lysates (Fig. 2h). Similar results were obtained with HEK293T (Fig. S1c) and A549 (Fig. S2c) cells. Deacetylation of 14-3-3ζ by histone deacetylase 6 (HDAC6) was previously shown to enhance its binding to client proteins [22]. α-Tubulin, another HDAC6 substrate, also showed an increased acetylation level in BA-treated BxPC-3 cells (Fig. S1d, S1e). However, BA did not directly inhibit HDAC6 activity in vitro (data not shown) or affect HDAC6 protein abundance in BxPC-3 cells (Fig. S1d, S1e). These results suggested that BA increases the acetylation level of 14-3-3ζ by a mechanism independent of HDAC6 and thereby attenuates the interaction between 14-3-3ζ and its client proteins, resulting in the dephosphorylation of these proteins by abundant intracellular phosphatases (Fig. 2i).

### BA inhibits H3S28 phosphorylation dependent on 14-3-3ζ

**Fig. 3:**
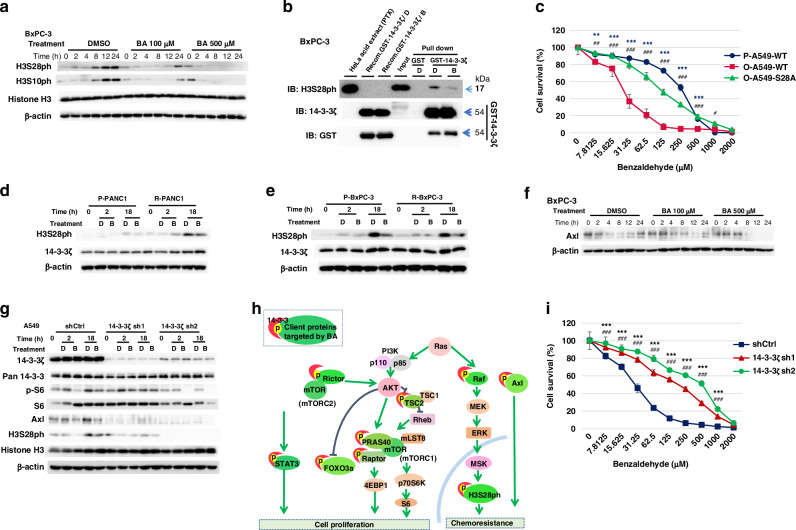
BA inhibits H3S28 phosphorylation dependent on 14-3-3ζ. **a** Immunoblot analysis of histone H3, H3S28ph, and H3S10ph in BxPC-3 cells treated with BA at 100 or 500 µM or with DMSO vehicle for the indicated times. **b** Pull-down assay with GST-tagged 14-3-3ζ (or GST) and high-salt extracts of BxPC-3 cells that had been treated with DMSO (D) or 1000 µM BA (B) for 100 min. The pull-down material as well as the original extracts (Input) were subjected to immunoblot analysis with antibodies to H3S28ph, 14-3-3ζ, and GST. The lane labeled HeLa acid extract (PTX: Paclitaxel treated) indicates positive control of H3S28ph, Recomb. GST-14-3-3ζ / D and Recomb. GST-14-3-3ζ / B indicate recombinant GST-14-3-3ζ with DMSO, and recombinant GST-14-3-3ζ with BA, respectively. **c** CellTiter-Glo assay for parental (P-) or osimertinib-resistant (O-) A549 cells expressing WT or S28A mutant forms of H3 and treated with the indicated concentrations of BA for 48 h. Data are means ± SD from *n* = 3 independent experiments. **p < 0.01, ***p < 0.001 for O-A549-WT versus O-A549-S28A; ^#^*p* < 0.05, ^##^*p* < 0.01, ^###^*p* < 0.001 for O-A549-WT versus P-A549-WT (two-tailed Student’s *t* test). Immunoblot analysis of H3S28ph and 14-3-3ζ in parental (P-) and radiation-resistant (R-) PANC1 (**d**) or BxPC-3 (**e**) cells treated with DMSO (D) or 1000 µM BA (B) for the indicated times. **f** Immunoblot analysis of Axl in BxPC-3 cells treated with BA at 100 or 500 µM or with DMSO vehicle for the indicated times. **g** Immunoblot analysis of the indicated proteins in A549 cells infected with lentiviruses encoding shRNAs (sh1 or sh2) specific for 14-3-3ζ or with the empty virus (shCtrl) and then treated with DMSO (D) or 500 µM BA (B) for the indicated times. **h** Schematic illustration of 14-3-3 proteins and their many client proteins regulated by BA related to signaling pathways activated in cancer. **i** CellTiter-Glo assay for 14-3-3ζ–depleted A549 cells as in (**g**) treated with the indicated concentrations of BA for 48 h. Data are means ± SD from *n* = 3 independent experiments. ***p < 0.001 for shCtrl versus 14-3-3ζ sh1, ^###^*p* < 0.001 for shCtrl versus 14-3-3ζ sh2.

The binding of 14-3-3ζ has been found to maintain the phosphorylation state of histone H3, which plays an important role in malignant transformation [11, 14]. Aurora B, a kinase that phosphorylates H3, has also been implicated in treatment resistance in NSCLC, with nonmitotic levels of phospho-H3 being increased by EGFR-TKI treatment [23]. Given that H3S28ph binds more strongly with higher affinity to 14-3-3ζ than does H3S10ph [9, 10, 24], we focused on H3S28ph, which may be influenced by 14-3-3ζ to a greater extent than H3S10ph. Treatment with BA at 100 or 500 µM markedly reduced the abundance of both H3S28ph and H3S10ph in BxPC-3 cells (Fig. 3a) and A549 cells (Fig. S2a), but not in (noncancerous) HEK293 cells (Fig. S2b). Given the inhibitory effect of BA on the interaction between 14-3-3ζ and its client proteins, we performed a pull-down assay with GST–14-3-3ζ and a high-salt extract of BxPC-3 cells containing H3S28ph tightly associated with nucleic acids. BA was shown to inhibit the interaction between GST–14-3-3ζ and H3S28ph (Fig. 3b).

To examine the possible role of H3S28ph in the increased sensitivity of osimertinib-resistant cells to BA, we introduced FLAG epitope–tagged wild-type (WT) or S28A mutant in which S28 is replaced with alanine forms of H3 into A549 cells by lentivirus infection(Fig. S2d). Osimertinib resistance was induced by exposure of the cells to the drug at 3 μM for 10 days. BA sensitivity was significantly decreased in the osimertinib-resistant cells expressing H3S28A (O-A549-28A cells) compared with those expressing wild-type H3 (O-A549-WT) (Fig. 3c), suggesting that the downregulation of H3S28ph by BA contributes to the suppression of the proliferation of EGFR-TKI–resistant cells by BA.

Immunoblot analysis showed that the abundance of H3S28ph was increased in radiation-resistant (R-) PANC1 and BxPC-3 cells compared with the corresponding parental (P-) cells and that H3S28ph was downregulated in these cells by BA treatment (Fig. 3d, e). These findings suggested that H3S28ph may influence BA sensitivity associated with radiation resistance.

Axl is also a client protein of 14-3-3ζ [25] and is implicated in osimertinib resistance [26]. BA treatment showed a delayed inhibitory effect on Axl expression in BxPC-3 (Fig. 3f) and A549 (Fig. S2a) cells, whereas a pull-down assay with GST-tagged 14-3-3ζ and A549 cell lysates showed that BA suppressed the interaction between GST–14-3-3ζ and Axl (Fig. S2c).

We directly examined the role of 14-3-3ζ in the growth-inhibitory effect of BA by depleting endogenous 14-3-3ζ in A549 cells with short hairpin RNAs (shRNAs). The BA-induced downregulation of phospho-S6 downstream of mTOR was attenuated by 14-3-3ζ shRNA2 but not by shRNA1, even though shRNA1 showed the greatest knockdown efficacy for 14-3-3ζ (Fig. 3g). Given that 14-3-3ζ functions as a dimer and may be replaced by other isoforms in markedly 14-3-3ζ–depleted cells [27], such replacement may have influenced the results of the shRNA experiments. Nevertheless, these results indicated that 14-3-3ζ is responsible, at least in part, for the suppression of phospho-S6 by BA. The inhibitory effects of BA on the abundance of H3S28ph and Axl were also attenuated in the 14-3-3ζ–depleted cells (Fig. 3g), implicating 14-3-3ζ in these effects of BA and reinforcing the notion that the suppression of tumor growth by BA is likely mediated by effects on multiple signaling pathways [6, 8] (Fig. 3h). Furthermore, we found that A549 cells depleted of endogenous 14-3-3ζ were less sensitive to the growth-inhibitory action of BA than were control cells (Fig. 3i), indicating that 14-3-3ζ is required for this action of BA.

### BA inhibits the transcription of multiple genes related to treatment resistance

**Fig. 4:**
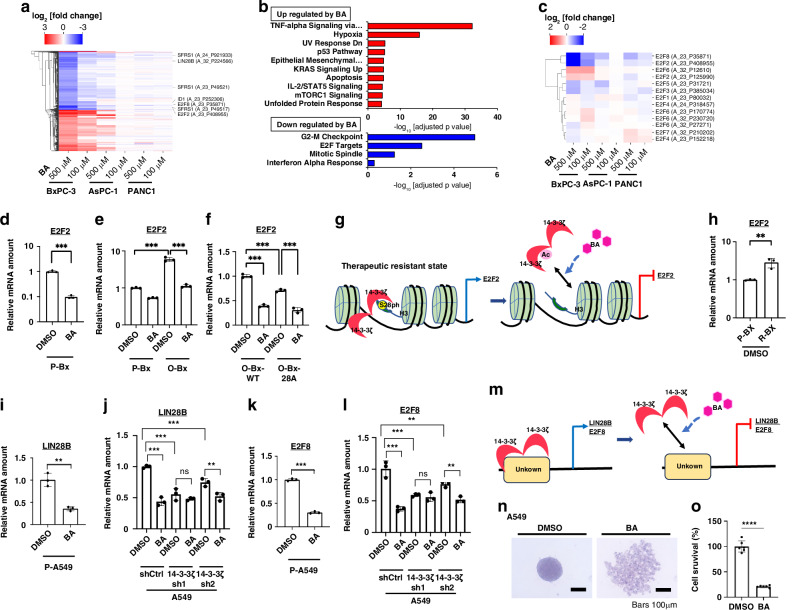
BA inhibits the transcription of multiple genes related to treatment resistance. **a** Hierarchical clustering of changes in gene expression for BxPC-3, AsPC-1, and PANC1 cells treated with BA at 100 or 500 μM for 24 h compared with DMSO-treated control cells, as determined by microarray analysis. The heat map includes genes that showed a log_2_[fold change] of ≥1 or ≤–1 in BxPC-3 cells treated with 500 μM BA. *SFRS1* (*SRSF1*), *LIN28B*, *ID1*, *E2F8*, and *E2F2* genes are labeled on the right side of the heat map with their respective probe set IDs. **b** Pathways upregulated (red bars, top) or downregulated (blue bars, bottom) by treatment with 500 µM BA in BxPC-3 cells. Bar length indicates –log_10_[adjusted *p* value]. **c** Heat map of changes in E2F family gene expression in the three cell lines treated with BA. The map shows log_2_[fold change] values. **d** RT-qPCR analysis of *E2F2* mRNA in parental (P-) BxPC-3 cells treated with 500 µM BA or DMSO for 20 h. **e** RT-qPCR analysis of *E2F2* mRNA in P-BxPC-3 and osimertinib-resistant (O-) BxPC-3 cells treated with 500 µM BA or DMSO for 20 h. **f** RT-qPCR analysis of *E2F2* mRNA in O-BxPC-3 cells expressing WT or S28A mutant forms of histone H3 and treated with 500 µM BA or DMSO for 20 h. **g** Schematic illustration of the proposed mechanism for regulation of *E2F2* transcription by H3S28ph under the stress condition induced by osimertinib treatment and for the effect of BA. **h** RT-qPCR analysis of *E2F2* mRNA in P-BxPC-3 and radiation-resistant (R-) BxPC-3 cells treated with DMSO for 20 h. **i** RT-qPCR analysis of *LIN28B* mRNA in P-A549 cells treated with 500 µM BA or DMSO for 20 h. **j** RT-qPCR analysis of *LIN28B* mRNA in A549 cells expressing 14-3-3ζ shRNAs or in control (shCtrl) cells treated with 500 µM BA or DMSO for 20 h. **k** RT-qPCR analysis of *E2F8* mRNA in A549 cells treated with 500 µM BA or DMSO for 20 h. **l** RT-qPCR analysis of *E2F8* mRNA in A549 cells as in **j**. All RT-qPCR data are means ± SD from three independent experiments. **p < 0.01,***p < 0.001; ns: not significant (two-tailed Student’s *t* test or one-way ANOVA followed by Tukey’s multiple-comparison test). **m** Schematic illustration of the proposed mechanism for regulation of the transcription of *LIN28B*, *E2F8*, and other genes by BA and 14-3-3ζ. Sphere-forming assay in A549 cells incubated with 500 µM BA or DMSO for 4 days: **n** Microscopic findings of the spheroid, **o** CellTiter-Glo assay (*n* = 6). ****p < 0.0001 (two-tailed Student’s *t* test).

To investigate the effects of BA on gene expression, we performed microarray analysis of three pancreatic cancer cell lines: BxPC-3 (high sensitivity to BA), AsPC-1 (moderate sensitivity), and PANC1 (low sensitivity). The analysis was conducted for cells treated with 0, 100, or 500 μM BA for 24 h. Cluster analysis was performed on genes showing more than a log2 fold change of ≥1 or ≤–1 in RNA expression (Fig. 4a). The changes in gene expression were greater in the highly BA-sensitive BxPC-3 cells than in the other two cell lines. Gene set enrichment analysis identified pathways significantly affected by treatment with 500 µM BA in BxPC-3 cells, including downregulation of the expression of E2F target genes (Fig. 4b). The E2F family of transcription factors is derived from eight genes and regulates important processes such as cell cycle progression, apoptosis, differentiation, DNA damage repair, metabolism, and angiogenesis [28]. Among E2F family members, expression of the genes for E2F2 and E2F8 was most notably suppressed by BA (Fig. 4c). E2F2 has been implicated in regulation of cancer stem cell (CSC) properties by various microRNAs [29–31] and necessary for self-renewal of stem cells and radioresistance of glioblastoma [32], whereas E2F8 is thought to be associated with tumorigenesis and radioresistance [33] as well as with EMT [34]. In addition, genes whose expression was suppressed by BA included *ID1*, which is associated with regulation of CSCs and treatment resistance [35]; *SRSF1*, which contributes to RNA splicing that alters cancer stem cell plasticity [36]; and *LIN28B*, an oncogenic stem cell factor involved in EMT [37] (Fig. 4a). Reverse transcription and quantitative polymerase chain reaction (RT-qPCR) analysis also showed that the abundance of *E2F2* mRNA was significantly reduced by BA treatment in both BxPC-3 (Fig. 4d) and A549 (Fig. S3a) cells, but it was significantly increased in noncancerous HEK293 (Fig. S3b).

H3S28ph has been implicated in gene transcription related to stress signaling [38]. We therefore examined whether H3S28ph-mediated transcriptional regulation might contribute to the emergence of treatment resistance, and whether the BA-induced downregulation of H3S28ph might explain the increased BA sensitivity of treatment-resistant cells. To this end, we generated parental (P-Bx) or osimertinib-resistant (O-Bx) BxPC-3 cells infected with lentiviruses encoding WT or S28A mutant forms of histone H3 (Fig. S3c). The O-Bx-S28A cells showed a reduced sensitivity to the growth-inhibitory effect of BA compared with O-Bx-WT cells (Fig. S3d). RT-qPCR analysis also revealed that *E2F2* expression was significantly increased in O-Bx cells compared with P-Bx cells, and that BA treatment significantly suppressed this increase (Fig. 4e). Furthermore, the abundance of *E2F2* mRNA was significantly lower in O-Bx-S28A cells relative to O-Bx-WT cells (Fig. 4f). These results suggested that *E2F2* transcription may be regulated by H3S28ph under the stress condition conferred by 10 days of osimertinib treatment (Fig. 4g). Of note, the extent of *E2F2* expression did not differ significantly between P-Bx-WT and P-Bx-S28A cells (data not shown).

Whereas *SRSF1* expression was significantly attenuated by BA treatment in O-Bx-WT cells, such an effect was not observed in O-Bx-S28A cells (Fig. S3e), suggestive of a role for H3S28ph in this action. The abundance of *E2F2* mRNA was not increased in osimertinib-resistant (O-) A549 cells compared with parental (P-) A549 cells, but it was significantly reduced in both cell lines by BA treatment (Fig. S3f). The level of *E2F2* expression was not significantly decreased in O-A549-28A cells relative to O-A549-WT cells, but the suppressive effect of BA on *E2F2* expression appeared somewhat attenuated in the former cells (Fig. S3g), suggesting that H3S28ph may be involved in BA-induced *E2F2* suppression. Whereas *SRSF1* expression was not significantly decreased in O-A549-S28A cells compared with O-A549-WT cells, the significant suppressive effect of BA apparent in the latter cells was not observed in the former (Fig. S3h).

Radiation-resistant BxPC-3 (R-BxPC-3) cells showed a significant increase in *E2F2* expression compared with parental (P-BxPC-3) cells (Fig. 4h). Expression of the gene for LIN28B, an RNA binding protein involved in EMT, was also suppressed by BA in A549 cells (Fig. 4i). The suppression of the growth-inhibitory effect of BA in A549 cells by depletion of endogenous 14-3-3ζ (Fig. 3g) was associated with attenuation both of *LIN28B* expression and of the inhibitory effect of BA on the expression of this gene (Fig. 4j).

Furthermore, the amount of *E2F8* mRNA in A549 cells was significantly reduced by BA treatment (Fig. 4k) as well as by knockdown of 14-3-3ζ, and the BA-induced suppression of *E2F8* expression in these cells was also attenuated by loss of 14-3-3ζ (Fig. 4l). Attenuation of the BA-induced suppression of *E2F2* and *SRSF1* expression by 14-3-3ζ knockdown was also evident in A549 cells (Fig. S3i, S3j). The expression of *ID1* was inhibited both by BA and by 14-3-3ζ knockdown (with sh1) in A549 cells, and this effect of BA was attenuated by depletion of 14-3-3ζ (Fig. S3k). Together, these findings suggested that 14-3-3ζ contributes to regulation of the transcription of *LIN28B*, *E2F8*, and other genes, and that BA suppresses the expression of these genes through 14-3-3ζ (Fig. 4m).

BA was shown to influence the transcription of genes involved in cancer stemness and EMT. Given the well-established link between stemness and EMT, we performed a sphere formation assay to evaluate stemness [39]. The formation of spheroids in A549 cells was markedly inhibited by treatment with 500 µM BA (Fig. 4n), and a significant reduction in cell viability was also observed (Fig. 4o). These findings demonstrate that BA suppresses stemness and EMT not only at the level of gene expression but also through in vitro experiments.

### BA inhibits pancreatic tumor growth, lung metastasis, and pleural dissemination

**Fig. 5:**
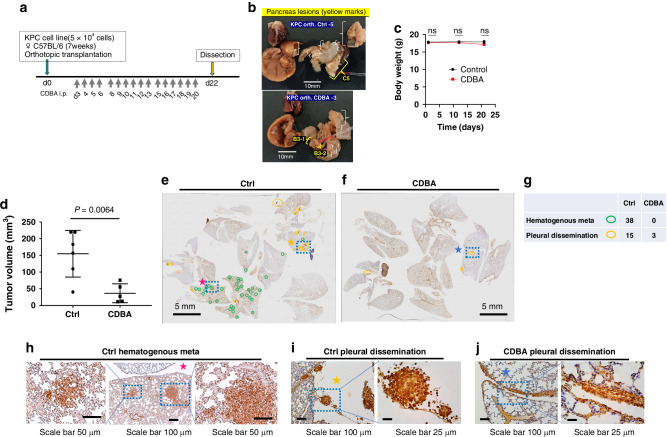
Treatment with the BA-derivative CDBA inhibits pancreatic tumor growth, lung metastasis, and pleural dissemination in a mouse model. **a** Time schedule of intraperitoneal (i.p.) CDBA treatment for C57BL/6 mice after orthotopic transplantation of the KPC cell line. **b** Anatomic findings for tumors of control (Ctrl) and CDBA-treated mice. **c** Time course of body weight for control (*n* = 6) and CDBA-treated (*n* = 5) mice. ns, not significant for comparisons between the two groups (two-tailed Student’s *t* test). **d** Calculated tumor volume for the pancreas of control and CDBA-treated mice. The mean ± SD values are indicated, and the *p* value was determined with the two-tailed Student’s *t* test. **e,** **f** Low-magnification images of immunohistochemical staining for EpCAM in lung sections of control and CDBA-treated mice. Green and yellow circles indicate hematogenous metastasis and pleural dissemination, respectively. **g** Counts for hematogenous metastases and pleural dissemination determined from the images in **e** and **f**. **h** High-magnification images of the boxed region marked by the pink asterisk in **e** showing hematogenous metastasis in the control lung. **i** High-magnification images of the boxed region marked by the yellow asterisk in **e** showing pleural dissemination forming a spheroid in the control lung. **j** High-magnification images of the boxed region marked by the blue asterisk in **f** showing pleural dissemination with multiple cell-cell contacts in the CDBA-treated lung.

The antitumor action of BA was evaluated in a mouse pancreatic cancer model. The orthotopic transplantation model [40] is based on transplantation of the KPC pancreatic cancer cell line derived from mice harboring Kras(G12D) and Trp53(R172H) mutations into 7-week-old female C57BL/6 mice [41]. The mice were treated with CDBA (β-cyclodextrin compound benzaldehyde) (Fig. S4a) administered intraperitoneally 6 days a week at a dose of 40 mg/kg for a total of 16 doses beginning 3 days after cell transplantation (Fig. 5a). Control and CDBA-treated mice underwent dissection at 22 days after cell transplantation for evaluation of the tumor-inhibitory effect of CDBA (Figs. 5b and S4b–S4g). Body weight did not differ significantly between control and CDBA groups during the treatment course (Fig. 5c). Although precise assessment of total tumor burden was challenging because of the presence of numerous disseminated microlesions in the peritoneal cavity, the tumor volume in the pancreas was significantly smaller for the CDBA group than for the control group (Fig. 5d). In addition, whereas multiple epithelial cell adhesion molecule (EpCAM)–positive lung metastases were detected in control mice, no such metastases were observed in CDBA-treated mice, and pleural surface dissemination was also less frequent in the latter animals (Fig. 5e–j).

### Effects of CDBA treatment on molecular and morphological characteristics of pancreatic tumors

**Fig. 6:**
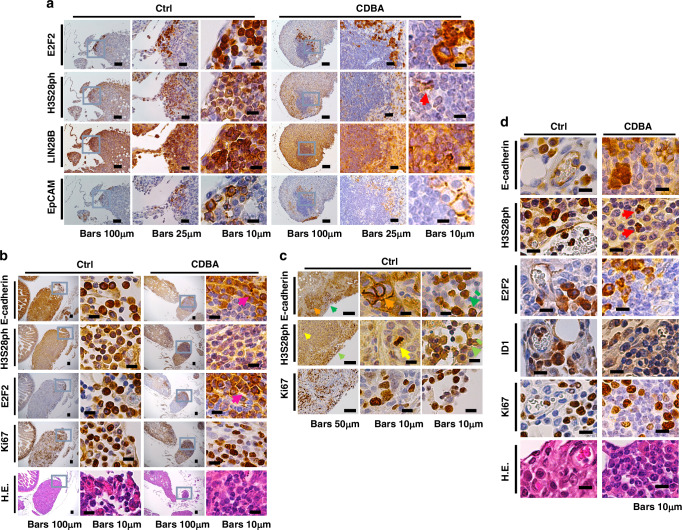
CDBA treatment induces morphological changes suggestive of a transition of EMP-like cells to the epithelial state in pancreatic tumors. **a** Immunohistochemistry of pancreatic tumor tissue from the mouse KPC orthotopic transplantation model. Serial sections of control (Ctrl) and CDBA-treated tumors were stained for E2F2, H3S28ph, LIN28B, and EpCAM. **b** Serial sections of control and CDBA-treated tumors were similarly stained for E-cadherin, H3S28ph, E2F2, and Ki67 as well as subjected to hematoxylin-eosin (H.E.) staining. **c** Serial sections of a control tumor were stained for E-cadherin, H3S28ph, and Ki67. **d** Serial sections of control and CDBA-treated tumors were stained for E-cadherin, H3S28ph, E2F2, ID1, and Ki67 as well as subjected to H.E. staining. Red arrows, cells manifesting mitotic catastrophe with damaged chromatin; orange arrows, canonical expression of E-cadherin at the cell membrane; green arrows, noncanonical E-cadherin expression in the cytoplasm of round EMP-like cells; yellow arrows, high H3S28ph expression associated with chromatin aggregation during mitosis; yellow-green arrows, mitosis-independent H3S28ph expression in round EMP-like cells; pink arrows, pearl formation by cells with epithelial features.

Immunohistochemistry was performed for serial sections of pancreatic tumor tissue from the KPC orthotopic transplantation model with antibodies to E2F2, H3S28ph, LIN28B, and other markers whose gene expression was suppressed by BA in vitro (Fig. 6a). In the control group, E2F2 was highly expressed in clusters of small, round cells located at the tumor margins and between tumor masses. The same regions in serial sections showed cell populations with a high abundance of H3S28ph and a high expression level of LIN28B. Cells that express LIN28B, which is implicated in EMT, often manifest mesenchymal characteristics [37]. In the regions of high LIN28B expression, the cell clusters highly expressing E2F2 also showed high expression of the epithelial marker EpCAM, which was present both at the cell membrane and in the cytoplasm. These findings suggested that these cell clusters possessed characteristics of epithelial-mesenchymal hybrid cells, or EMP. E2F2-positive cells were also present near the tumor margins in the CDBA treatment group. These cells were located primarily at the periphery of clusters of small, round lymphocyte-like cells and appeared deformed, with E2F2 being frequently detected in the cytoplasm rather than the nucleus. Analysis of serial sections revealed that the abundance of H3S28ph was markedly reduced in the E2F2-positive regions compared with control mice, with H3S28ph retention being apparent in deformed nuclei suggestive of mitotic catastrophe (red arrows in Figs. 6a and S5). LIN28B expression in the nucleus of cells was also markedly reduced by CDBA treatment. EpCAM was expressed at a low level at the cell membrane of cells with enlarged, deformed cytoplasm in the E2F2-positive regions of CDBA-treated tumors, representing a phenotype different from that of control tumors.

We also performed immunohistochemical analysis of serial sections with antibodies to E2F2, H3S28ph, E-cadherin, and Ki67 (Fig. 6b). Relocalization of adhesion proteins such as E-cadherin from the cell surface to the cytoplasm has been identified as an early feature of EMT in epithelial-mesenchymal hybrid cells involved in cancer cell migration and metastasis [42]. Examination of E-cadherin staining in the control group revealed the canonical phenotype of high expression at the cell surface in areas with clusters of large tumor cells (Fig. 6c, orange arrows). However, at the tumor margins, localized clusters of small, round cells with high expression of E-cadherin but lacking polarity were identified (green arrows). Furthermore, in the regions containing large tumor cells, H3S28ph was highly expressed in mitotic (Ki67-positive) cells showing chromatin condensation (yellow arrows). On the other hand, high mitosis-independent expression of H3S28ph was apparent in the clusters of round cells with high E-cadherin expression that had lost their polarity (yellow-green arrows). Ki67 was highly expressed in many cells within both cell groups, indicative of ongoing cell division (Fig. 6b, c).

These findings suggested that the clusters of small, round cells in control mice likely represent stem-like EMP cells. Further immunostaining of serial sections revealed high expression of ID1, which plays a role in asymmetric division [43], of the stem cell marker CD44v6 [44], and of Cre recombinase (marker for the transplanted cells) in the clusters of small, round tumor cells of control mice (Figs. 6d and S6). In addition, these cell clusters were often located adjacent to newly forming blood vessels (Fig. 6d).

E2F2 expression was not observed in the bulk tumor areas with the exception of vessel walls, and it colocalized with E-cadherin expression in the small, round tumor cells of control mice, suggestive of a high specificity as a marker of EMP cells. In the CDBA-treated group, E2F2-positive cells were observed in the regions with cytoplasmic E-cadherin expression, but these cells showed an increased number of cell junctions and layered structures resembling pearl formations (pink arrows in Figs. 6b and S6). To quantify these observations, we marked EMP-like cell clusters with high expression of cytoplasmic E-cadherin and E2F2 on pancreatic tissue sections from mice. These clusters were outlined with circles, while those exhibiting morphological changes induced by CDBA treatment were outlined with triangles. Both types were subsequently counted (Figs. S7 and S8). Furthermore, we identified clusters of cells with mitosis-independent, high H3S28ph expression colocalized with E2F2-positive regions, marking their locations as well (Fig. S9). These results showed that the number of EMP-like cell clusters decreased in CDBA-treated mice compared to controls, indicating that CDBA treatment attenuated the epithelial-mesenchymal hybrid state.

Examination of additional histone modifications revealed that the activation marker H3K4me3 (histone H3 trimethylated at lysine-4) was highly expressed in tumor cells of control mice, but that its expression was not substantially affected by CDBA treatment (Fig. S10a). The mitotic marker H3S10ph was expressed at a low level in EMP-like cells of control mice, with its expression being attenuated by CDBA treatment, albeit not to the same extent as for H3S28ph (Fig. S10b).

Round cells in the peritoneal dissemination state showed high E-cadherin expression and high mitotic-independent H3S28ph expression for control tumors (Fig. S11). Clusters of spherical cells at the pleural surface were found to be EpCAM-positive, indicative of migratory capacity associated with the EMP state. These cells appeared to be derived from membrane-like structures near the pleural surface (Fig. 5i). Small accumulations of cells in the liver of control mice were positive for EpCAM, H3S28ph, 14-3-3ζ, and ID1 (Fig. S12), suggesting that they were metastases. Such accumulations were less common in the CDBA-treated group (data not shown).

**Fig. 7:**
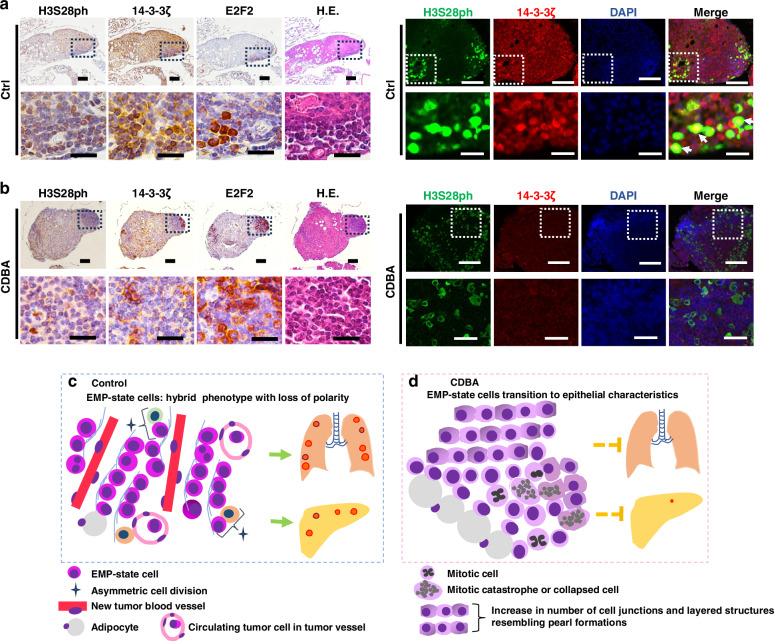
CDBA treatment induces morphological changes of EMP-like cells positive for H3S28ph, 14-3-3ζ, and E2F2 suggestive of epithelial transition in pancreatic tumors. **a,** **b** Immunohistochemical (left) and immunohistofluorescence (right) staining of serial sections of pancreatic tumor tissue from the mouse KPC orthotopic transplantation model. Immunohistochemical staining for H3S28ph, 14-3-3ζ, and E2F2 is shown together with H.E. staining. Immunofluorescence staining for H3S28ph and 14-3-3ζ is shown together with DAPI staining. The tissue was derived from control (**a**) or CDBA-treated (**b**) mice. White arrows indicate colocalization of H3S28ph and 14-3-3ζ. Scale bars: 100 and 25 µm (top and bottom images of each pair, respectively). **c,** **d** Schematic interpretation of morphological findings. Control tumors are characterized by the presence of epithelial-mesenchymal hybrid (EMP) cells, active neovascularization, intravascular dissemination, and multiorgan metastasis (**c**), whereas the expression of epithelial-mesenchymal hybrid markers was attenuated in CDBA-treated tumors in association with a shift in phenotype to the epithelial state and inhibition of metastasis (**d**).

We also performed immunofluorescence staining of H3S28ph and 14-3-3ζ as well as nuclear staining with 4′,6-diamidino-2-phenylindole (DAPI) in serial sections corresponding to immunohistochemical staining of E2F2, H3S28ph, and 14-3-3ζ as well as hematoxylin-eosin (H.E.) staining for both control and CDBA-treated tumors (Fig. 7a, b). Areas showing colocalization of H3S28ph, 14-3-3ζ, and DAPI staining were apparent in control tumors (white arrows), and CDBA treatment markedly reduced the expression of both 14-3-3ζ and H3S28ph in such regions.

Summarizing our immunostaining findings for the control group, localized clusters of round cells that expressed E-cadherin at a high level but had lost polarity were detected at the tumor margins (Figs. 7c and S7). These cells also showed high expression of EpCAM, LIN28B, and ID1, suggestive of an epithelial-mesenchymal hybrid (EMP) state and the acquisition of migratory capacity. E2F2, whose expression at the transcriptional level is regulated by H3S28ph, was also specifically expressed in this group of cells. These cells underwent cell division and were associated with active neovascularization, and many cells with a similar expression pattern were detected within blood vessels, suggesting that they contribute to intravascular dissemination and multiorgan metastasis. Similar cell clusters were also observed in peritoneal and pleural disseminations. In the case of the CDBA group, CDBA treatment reduced the expression of epithelial-mesenchymal hybrid markers in the corresponding cell populations, shifting the phenotype of the cells toward the epithelial state, with an increased number of cell junctions and differentiation from loosely adherent round cells to tightly connected cells (Fig. 7d). Neovascularization was less prominent, and the number of circulating tumor cells in blood vessels was decreased, in the CDBA-treated mice, consistent with the suppression of lung metastasis.

## Discussion

We demonstrated that BA disrupts the interaction between 14-3-3ζ and its client proteins, leading to reduced phosphorylation and suppression of cancer-related signaling pathways. Furthermore, we identified H3S28ph, a client of 14-3-3ζ, as a key factor in the mechanism by which sensitivity to the cytotoxic action of BA is increased in cancer cells resistant to radiation or osimertinib treatment. H3S28ph or 14-3-3ζ or both played a role in BA’s suppression of CSC- and EMT-related genes like E2F2, ID1, SRSF1, LIN28B, and E2F8.

Experiments with the mouse KPC orthotopic transplantation model of pancreatic cancer positive for *Kras* and *Trp53* mutations revealed a localized cell population around and within blood vessels that manifested epithelial-mesenchymal hybrid characteristics, including a loss of polarity associated with cytoplasmic expression of E-cadherin and EpCAM as well as expression of ID1, CD44v6, and LIN28B. This cell population also showed mitosis-independent expression of H3S28ph as well as colocalization of E2F2 and 14-3-3ζ. We considered these clusters of small, round cells to be in a state of EMP and therefore likely to possess increased migratory and metastatic capacities. Treatment of the tumor-bearing mice with BA shifted the morphology of these cells toward an epithelial phenotype and inhibited lung metastasis.

Members of the 14-3-3 protein family interact with numerous targets involved in key cellular processes such as signal transduction, apoptosis, cell cycle regulation, and cell migration. These 14-3-3 isoforms serve to prevent the dephosphorylation of phosphorylated sites by protein phosphatases through interaction with these sites in client proteins. Expression of the 14-3-3ζ isoform is increased in many cancer types and is associated with poor prognosis as a result of its links to metastasis, recurrence, and treatment resistance [6–8]. This isoform has also been implicated as a hub for stress adaptation signaling in cancer [8]. In addition, 14-3-3 proteins function as cruciform DNA binding proteins [45] and may contribute to DNA replication and transcription [46, 47].

H3S28ph, a client protein of 14-3-3ζ, serves as a mitotic marker together with H3S10ph. The phosphorylation of histone H3 is associated with chromatin condensation mediated by Aurora B during mitosis, but it is also implicated in mitosis-independent transcriptional regulation during interphase. H3S28ph is upregulated in Ras-transformed cells and also plays a role in transcriptional processes induced by the tumor-promoting phorbol ester TPA [11]. In addition, it contributes to epidermal growth factor (EGF)–induced tumorigenic transformation mediated by MSK1 [14], and its abundance is increased by ultraviolet-B radiation through MSK1 [48]. H3S28ph has been found to be associated with the H3 variant H3.3 present in transcriptionally active chromatin as well as with unstable nucleosomes [49, 50]. It mediates the dissociation of the polycomb repressive complex PRC2 from neighboring H3K27 [51, 52]. The observed enrichment of H3S28ph marks at promoters and 5’ untranslated regions (with 53% overlapping with CpG islands) further suggests an involvement with polycomb complex function and implicates H3S28ph in cell fate determination [38]. Mitosis-independent phosphorylation of H3 has also been implicated in treatment resistance in NSCLC [23]. Induction of locus-specific endogenous histone phosphorylation by MSK1 has recently been made possible with the use of the CRISPR/Cas9 system, and has revealed a causal relation between histone phosphorylation and promoter activation, with H3S28ph playing a key role in this process [53].

H3S28ph does not bind to 14-3-3ζ during mitosis, but its phosphorylation state is maintained through interaction with 14-3-3ζ during interphase, which promotes transcriptional activity [9, 10]. This binding of 14-3-3ζ to H3S28ph occurs with a higher affinity than that between 14-3-3ζ and H3S10ph [9, 10, 24]. In addition, knockdown of 14-3-3ζ was shown to sensitize EGFR-TKI–resistant human lung adenocarcinoma cells to gefitinib and to inhibit EMT [54]. These various findings implicate H3S28ph and 14-3-3ζ in malignant transformation and EGFR-TKI treatment resistance. We have now shown that BA disrupts the interaction between H3S28ph and 14-3-3ζ, thereby reducing the phosphorylation level of H3S28 and suppressing the expression of many genes related to EMT, treatment resistance, and stemness. Our findings thus suggest the operation of an epigenetic control mechanism mediated by H3S28ph and 14-3-3ζ in treatment resistance established through treatment-induced stress responses.

In cancer, EMT is associated with tumor initiation, invasion, metastasis, and resistance to therapy. Cancer cells undergoing metastasis manifest an epithelial-mesenchymal hybrid phenotype as a result of cellular plasticity now referred to as EMP [17, 18]. Key criteria for defining EMP in epithelial cells include changes in cell characteristics such as the loss of cell junctions and increased migratory capacity [16]. The most mesenchymal-like cell populations in tumors are localized near endothelial and inflammatory cells, and they secrete chemokines and other proteins that attract immune cells and promote angiogenesis, and thereby orchestrate formation of their own inflammatory and highly angiogenic niche [55]. The loss of E-cadherin at the cell membrane and an increase in its intracellular abundance are characteristics of EMP [42]. We identified such an EMP-like cell population in the KPC orthotopic transplantation mouse model and we propose that E2F2, which was found to be largely localized to cells with cytoplasmic E-cadherin, is a specific marker for the stage of metastasis acquisition in EMP.

DNA methylation and histone H3 modifications have been implicated in the regulation of EMP [19, 20]. We found that mitosis-independent H3S28ph was localized to cells expressing E2F2 at a high level in mouse pancreatic tumors and that it contributed to *E2F2* expression in stressed cells in vitro. These results suggest that H3S28ph may be an important epigenetic regulatory factor in EMP. BA treatment of pancreatic tumors was found to attenuate the expression or change the intracellular distribution of factors highly expressed in the EMP-like cells, leading to a shift toward an epithelial phenotype associated with increased cell junctions and reduced lung metastasis and pleural dissemination.

The 14-3-3ζ protein has long been considered a target for cancer therapy, but its direct inhibition is problematic as a result of its various important functions in normal cells. Our results now suggest that BA, which is the simplest aromatic aldehyde and inhibits the interaction between 14-3-3ζ and client proteins, has the potential to overcome the problem of treatment resistance in cancer therapy and to inhibit EMP associated with metastasis.

## Materials and methods

### Cell culture

All human cell lines were cultured in RPMI 1640 medium (Sigma-Aldrich) or Dulbecco’s modified Eagle’s medium (DMEM, Nacalai Tesque) supplemented with 10% fetal bovine serum, penicillin (100 U/ml), and streptomycin (100 U/ml). All cells were maintained at 37 °C under 5% CO_2_ and at 100% humidity. The culture plates were sealed to prevent evaporation during the treatment of cells with BA (Sigma-Aldrich) or DMSO vehicle.

### Pathway analysis

Pathway analysis for genes that were found to be up- or downregulated in response to BA treatment in BxPC-3 cells by microarray analysis was performed with the Enrichr platform (https://maayanlab.cloud/Enrichr) [56, 57].

### Statistical analysis

For the Cell Titer-Glo assay, colony formation assay, sphere formation assay, and RT-qPCR analysis, with equal sample sizes and assuming 90% power and a significance level of 5%, we calculated that at least three independent experiments were required for each group to detect a difference between conditions. No data were excluded from the analyses. For all experiments, samples were randomly allocated into experimental groups whenever possible. Investigators were blinded to group allocation during data analysis for all experiments involving qualitative analysis of data from more than one group. Quantitative data are presented as means ± SD unless indicated otherwise. Variance was assessed and found to be similar between the groups being statistically compared. A two-tailed Student’s *t*-test was used for comparisons between two groups, and a one-way analysis of variance (ANOVA) followed by Tukey’s post hoc test was applied for comparisons among three or more groups. A *p*-value of <0.05 was considered statistically significant. All statistical analyses were performed using GraphPad Prism software.

### Other methods

All other methods are included in Supplementary Information.

## Supplementary information

Supplementary Information

## Data Availability

Microarray data are available in the GEO database under the accession number GSE275246.
