# Supplementary material for: Benzaldehyde suppresses epithelial-mesenchymal plasticity and overcomes treatment resistance in cancer by targeting the interaction of 14-3-3ζ with H3S28ph
Source: Br J Cancer. 2025 May 2;133(1):27–39. doi: 10.1038/s41416-025-03006-4 (PMC12238390; doi:10.1038/s41416-025-03006-4)
Supplement: Supplementary file 1 — Supplementary Information [file 41416_2025_3006_MOESM1_ESM.docx]

# **List of Supplementary Information**

List Supplementary Materials and Methods

- Cell lines
- In vitro assay of cell viability (XTT assay)
- In vitro assay of cell proliferation (CellTiter-Glo assay)
- HDAC6 activity assay
- Establishment of osimertinib-resistant cells
- Establishment of radiation-resistant cells
- Colony formation assay
- Immunoblot analysis
- Immunoprecipitation
- Pull-down assay
- Lentiviral infection
- Microarray analysis
- RT-qPCR analysis
- Sphere formation assay
- Animal experiments
- Immunohistochemical staining
- Immunohistofluorescence staining

List Supplementary Tables

- Supplementary Table 1. 　Antibody information
- Supplementary Table 2. 　Primer information

List Supplementary Figures

- Supplementary Figure 1.
- Supplementary Figure 2.
- Supplementary Figure 3.
- Supplementary Figure 4.
- Supplementary Figure 5.
- Supplementary Figure 6.
- Supplementary Figure 7.
- Supplementary Figure 8.
- Supplementary Figure 9.
- Supplementary Figure 10.
- Supplementary Figure 11.
- Supplementary Figure 12.

# **Supplementary Materials and Methods**

**Cell lines**

BxPC-3, A549, PANC1, AsPC-1, Capan-1, Capan-2, MIA Paca2, NCI-H23, U251MG, U87MG, DAOY, HeLa, BT474, MCF7, DU145, PC3, AG-S, HT29, A431, IMR-90, Hs742.Sk, ARPE19, HEK293, and HEK293T cell lines were obtained from American Type Culture Collection, HCT116 from RIKEN BRC, MKN28 and TIG-1-20 from JCRB Cell Bank, MRC-9 from Sigma-Aldrich, Hs 1.Tes and PNT2 from KAC Co. Ltd., SAEC from Lonza Bioscience, and Lenti-X 293T from Takara Bio. All cells were authenticated by STR profiling and tested for mycoplasma contamination.

**In vitro assay of cell viability (XTT assay)**

Cells were seeded in 96-well plates (3000 cells per well) and cultured overnight. BA was then added at various concentrations, and the cells were incubated for 72 h before assessment of cell viability with the use of a Cell Proliferation Kit II (XTT) (Merck, Roche) and a plate reader (EnVision, Perkin-Elmer) and calculation of IC_50_ values.

**In vitro assay of cell proliferation (CellTiter-Glo assay)**

Cells were seeded in 96-well plates at a density of 2000 cells per well for PANC1, 2500 cells per well for A549, and 3000 cells per well for BxPC-3, and were then cultured overnight. They were exposed to drugs at various concentrations for 48 h before assessment of cell viability with the use of a CellTiter-Glo Luminescent Cell Viability Assay Kit (Promega) and a plate reader.

**HDAC6 activity assay**

HDAC6 enzyme activity was measured with the use of a Fluorogenic HDAC6 Assay Kit (Bioscience).

**Establishment of osimertinib-resistant cells**

A549 and BxPC-3 cells were cultured for 10 days in medium supplemented with 3 µM osimertinib (Selleck Chemicals) in order to generate osimertinib-resistant (O-A549 and O-BxPC-3) cells.

**Establishment of radiation-resistant cells**

X-irradiation was performed with an MBR-1520R-4 system (Hitachi Power Solutions) set at 150 kV and 20 mA. The dose rate of radiation was 1.45 Gy/min. For establishment of radiation-resistant (R-) cells, PANC1 (1.2 × 10^6^) and BxPC-3 (2.5 × 10^6^) cells were seeded in 10-cm dishes to ~40% confluency and irradiated at 2 Gy/day for 5 days/week for a total dose of 50 Gy.

**Colony formation assay**

Parental or radiation-resistant PANC1 cells were seeded at a density of 300 cells per 6-cm dish, cultured for 2 h, and exposed to 2 Gy of x-radiation or 1200 µM BA (or both). After culture for 14 days, the cells were fixed with 4% paraformaldehyde and stained with 0.2% crystal violet (Sigma-Aldrich). Images of the dishes were captured for counting of colonies with the use of the Analyze Particles function of ImageJ software.

**Immunoblot analysis**

Cells were seeded at a density of 1.5 × 10^5^ to 2.5 × 10^5^ cells per well in six-well plates, cultured for 20 h, and then exposed for the indicated times to fresh medium containing various concentrations of BA. The cells were then washed with phosphate-buffered saline (PBS), lysed in SDS sample buffer (50 mM Tris-HCl [pH 6.8], 10% SDS, 5% glycerol, 5% β-mercaptoethanol), and subjected to immunoblot analysis. Primary antibodies are listed in Table S1. Immune complexes were detected with the use of a Western Lightning Plus, Chemiluminescent Substrate (PerkinElmer) and a LAS-3000 system (FujiFilm).

**Immunoprecipitation**

HEK293T cells were seeded at a density of 2.5 × 10^5^ cells per well in six-well plates, cultured for 24 h, and transfected with 9 µg of cDNA vector encoding Myc epitope–tagged human 14-3-3 isoforms　 with the corresponding empty vector with the use of the FuGENE HD reagent (Promega). After culture for 24 h, the cells were exposed to 500 µM BA or DMSO vehicle for an additional 24 h. They were then washed with PBS, detached by exposure to trypsin, and counted. The cells (1 × 10^7^) were lysed for 30 min at 4°C in 1 ml of immunoprecipitation lysis buffer (100 mM Tris-HCl [pH 7.7 ], 300 mM NaCl, 10 mM EDTA, 2% Nonidet P-40) supplemented with PhosSTOP (Sigma-Aldrich) and cOmplete Mini (Sigma-Aldrich) phosphatase and protease inhibitors, respectively. The lysate was centrifuged to remove debris, 50 µl of the resulting supernatant were saved as the input sample, and 900 µl of the supernatant were subjected to immunoprecipitation at 4°C with rotation for at least 2 h and with 5 µl of antibodies to the Myc epitope tag (Table S1) and protein A beads that had been pretreated with bovine serum albumin to block nonspecific sites. The beads were then washed before elution of immunoprecipitated proteins with a buffer containing 50 mM Tris-HCl [pH 6.8], 2% SDS, and 10% glycerol followed by immunoblot analysis with antibodies to 14-3-3 binding proteins (Table S1).

**Pull-down assay**

Recombinant GST-tagged human 14-3-3ζ protein (GST-14-3-3ζ) was prepared, and HEK293T, BxPC-3, or A549 cells were incubated with DMSO or 1000 µM BA for 100 min and lysed as for immunoprecipitation described above. The lysates were cleared of debris and incubated for 30 min at 4°C with Glutathione Sepharose™ 4B 50 µL (GE Healthcare) and 1 µg GST-14-3-3ζ. The beads were then washed before elution of bound proteins with a buffer containing 50 mM Tris-HCl [pH 6.8], 2% SDS, and 10% glycerol followed by immunoblot analysis with antibodies to 14-3-3ζ binding proteins (Table S1). For detection of the interaction between GST–14-3-3ζ and either H3S28ph or Axl, cells were lysed in a high-salt buffer (100 mM Tris-HCl [pH 7.7], 1 M NaCl, 10 mM EDTA, 2% Nonidet P-40) supplemented with PhosSTOP and cOmplete Mini. HeLa acid extract An extract (PTX: paclitaxel-treated) (Active Motif) was used as a positive control for the H3S28ph band.

**Lentiviral infection**

The vectors pLV-Puro-FLAG-hH3F3A or -hH3F3A-S28A encoding FLAG-tagged WT or S28A mutant forms of human histone H3 were designed and produced by VectorBuilder (VB190409-1161cwr and VB190409-1173fmr, respectively). The pLKO-puro-shRNA vectors for 14-3-3ζ sh1 (TRCN0000029405) or 14-3-3ζ sh2 (TRCN0000029406) were obtained from Sigma-Aldrich, with the empty pLKO-puro vector being used as shCtrl. These various lentiviral vectors together with psPAX2 (Addgene) and pCMV-VSV-G (Takara Bio) were introduced into Lenti-X 293T cells by transfection with the use of the FuGENE HD reagent (Promega). Culture supernatants containing lentiviruses were then applied to infection of A549 or BxPC-3 cells, and stable clones were selected in medium containing puromycin.

**Microarray analysis**

BxPC-3, AsPC-1, and PANC1 cells were cultured for 24 h with or without 100 or 500 µM BA, after which total RNA was extracted from the cells with the use of an RNeasy Mini Kit (Qiagen). The RNA samples were labeled with the use of a Quick Amp Labeling Kit (Agilent Technologies), and the labeled cRNA was allowed to hybridize to an oligonucleotide microarray (Whole Human Genome 4×44K, Agilent Technologies) for 17 h at 65°C. The slides were washed with the use of a Gene Expression Wash Buffer Kit (Agilent Technologies), dried, and scanned with an Agilent Microarray Scanner. The data were analyzed with Feature Extraction software version 9.5.1 (Agilent Technologies). Normalization was performed by global-normalization methods. Clustering analysis and visualization were performed with the use of Python libraries including matplotlib and seaborn.

**RT-qPCR analysis**

BxPC-3 cells (1.8 × 10^5^/well) and A549 cells (1.5 × 10^5^/well) were seeded in six-well plates, cultured for 20 h, and treated with DMSO or 500 µM BA for 20 h. Total RNA was isolated from the cells with the use of the TRIzol reagent (Invitrogen) and treated with DNase I. Portions of the RNA (500 ng) were subjected to RT with the use of a Two-Step cDNA Synthesis Kit (Roche), and the resulting cDNA was subjected to real-time PCR analysis in triplicate with SYBR Premix Ex Taq II (Takara Bio) and a Thermal Cycler Dice TP800 instrument (Takara Bio). Data were normalized by the abundance of *HPRT1* mRNA. The PCR primer sequences are listed in Table S2.

**Sphere formation assay**

A549 cells were seeded into Costar Ultra Low Cluster 96-well plates (Corning) at a density of 2000 cells per well in RPMI1640 medium containing 10% serum. For the BA treatment group, 500 μM of BA was added 2 hours after seeding, while DMSO was added to the control group. Each well was sealed with a plate seal to prevent evaporation and incubated in a humidified incubator. After 4 days, spheroids were observed under a microscope, and photographs were taken. Cell viability was then measured using the Cell Titer-Glo assay, and the number of viable cells was quantified using a plate reader (n=6). Statistical analysis was performed to assess significance.

**Animal experiments**

All animal experiments were approved by the Keio University Animal Care Committee (approval no. 19036) as well as the Recombinant DNA Experiment Committee (protocol no. D2019-013, registration no. D000239), and they were conducted in accordance with established guidelines.

KPC cells (C57BL/6 genetic background, Pdx1-Cre, lox-stop-lox-Kras(G12D)/+, lox-stop-lox-Trp53(R172H)/+; catalog no. 153474, Cancer Research UK, Glasgow Beatson Institute) [40] were implanted orthotopically into the pancreas [39] of 12 female C57BL/6 mice (CLEA Japan) at 7 weeks of age. No statistical test was performed to predetermine sample size for animal studies. Mice were randomly allocated to experimental groups whenever possible. The procedure was performed by laparotomy with animals anesthetized by Domitor (Zenoaq) 0.3 mg/kg + Midazoramu (SANDOZ) 4 mg/kg + Butorphanol tartrate (Meiji Seika) 5mg/kg. A total of 5 × 10^4^ cells was transplanted per mouse. The 12 mice were divided into four groups of three. Three days after cell implantation, two groups were randomly selected as treatment groups by a third party and received intraperitoneal injections of CDBA (persionally ordered Shiratori Pharmaceutical Co.) at a dose of 40 mg/kg per day for 6 days a week and a total of 16 doses. Body weight was measured on days 1, 12, and 22. On day 22, five mice from the CDBA treatment groups (one mouse died at 4 days after the onset of treatment) and six mice from the control groups were killed for isolation of tumor tissue and determination of pancreatic tumor volume. The excised tissue was fixed with 4% paraformaldehyde, embedded in paraffin, sectioned at a thickness of 4 μm, and either stained with hematoxylin and eosin (H.E.) or subjected to immunohistochemical or immunohistofluorescence analysis.

**Immunohistochemical staining**

Tissue sections were incubated with primary antibodies (Table S1) for 12 h at 4°C, and immune complexes were detected with the use of a Vectastain Elite ABC-HRP Kit (Vector Labs) and a Mouse on Mouse Immunodetection Kit (Vector Labs). The sections were then observed with a Nikon ECLIPSE Ni microscope.

**Immunohistofluorescence staining**

Tissue sections were depleted of paraffin and incubated with primary antibodies (Table S1) for 12 h at 4°C. They were then washed three times with PBS and exposed for 1 h at room temperature to appropriate Alexa Fluor 488– or Alexa Fluor 555–labeled secondary antibodies (Life Technologies) diluted in PBS. Nuclei were stained with Vectashield Vibrance Antifade Mounting Medium with DAPI (Vector Labs), and the sections were observed with a Biorevo BZ-9000 fluorescence microscope (Keyence).

**Supplementary Tables**

| **Supplementary Table 1：Antibodies information** | | |  |
| --- | --- | --- | --- |
| **Molecule** | **Cat. No.** | **Company** | **Application** |
| Phopho-p70 S6 Kinase(Thr389) (108D2) Rabbit mAb | 9234 | Cell Signaling | WB |
| p70 S6 Kinase Antibody | 9202 | Cell Signaling | WB |
| Phospho-S6 Ribosomal Protein(Ser235/236) Rabbit mab | 4858 | Cell Signaling | WB |
| S6 Ribosomal Protein(5G10) Rabbit mAb | 2217 | Cell Signaling | WB |
| Phospho-4E-BP1(T37/46) Rabbit mAb | 2855 | Cell Signaling | WB |
| 4EBP1(53H11) Rabbit mAb | 9644 | Cell Signaling | WB |
| Phosph-AKT(Ser473)(D9E)XE Rabbit mAb | 4060 | Cell Signaling | WB |
| AKT antibody | 9272 | Cell Signaling | WB |
| Phospho-Stat3(Tyr705)(D3A7)XP Rabbit mAb | 9145 | Cell Signaling | WB |
| Stat3(79D7) Rabbit mAb | 4904 | Cell Signaling | WB, IP |
| Phospho-p44/42MAPK (Erk1/2)(Yhr202/Tyr204)(D13.14.4E)XP Rabbit mAb | 4370 | Cell Signaling | WB |
| p44/42 MAPK(Erk1/2) Antibody | 9102 | Cell Signaling | WB |
| Monoclonal Anti-beta-Actin antibody produced in mouse | A5441 | SIGMA | WB, IP |
| Phospho-FoxO3a(Ser253) Antibody | 9466 | Cell Signaling | WB |
| FoxO3a(75D8) Rabbit mAb | 2497 | Cell Signaling | WB, IP |
| Phospho-FoxO1(Thr24)/FoxO3a(Thr32)/foxO4(Thr28) (4G6) Rabbir mAb | 2599 | Cell Signaling | WB |
| FoxO1(C29H4) Rabbit mAb | 2880 | Cell Signaling | WB, IP |
| Phospho PRAS40 | 2640 | Cell Signaling | WB |
| PRAS40 Antibody | 2610 | Cell Signaling | WB, IP |
| Phopho-Tubelin/TSC2(Ser939) Antibody | 3615 | Cell Signaling | WB |
| Tuberin/TSC2 Antibody | 3612 | Cell Signaling | WB, IP, PD |
| Phospho-Rictor(Thr1135)(D3OA3) Rabbit mAb | 3806 | Cell Signaling | WB |
| Rictor (53A2) Rabbit mAb | 2114 | Cell Signaling | WB, IP, PD |
| Phospho-c-Raf(Ser338)(56A6) Rabbit mAb | 9427 | Cell Signaling | WB |
| c-Raf Antibody | 9422 | Cell Signaling | WB, IP |
| NF-κB p65 (F6) Antibody | sc-8008 | Santa Cruz Biotechnology | WB |
| Phospho-(Ser) 14-3-3 Binding Motif Antibody | 9601 | Cell Signaling | WB |
| 14-3-3ζ Antibody (C-16) | sc-1019 | Santa Cruz Biotechnology | WB, IP, IF |
| 14-3-3ζ polyclonal antibody | BML-SA482 | Enzo | IHC |
| 14-3-3β Antibody (C-20) | sc-628 | Santa Cruz Biotechnology | WB, IP |
| 14-3-3ε Antibody (T-16) | sc-1020 | Santa Cruz Biotechnology | WB, IP |
| 14-3-3η Antibody | 18645 | IBL | WB, IP |
| 14-3-3γ Antibody (C-16) | sc-731 | Santa Cruz Biotechnology | WB, IP |
| 14-3-3θ Antibody (C-17) | sc-732 | Santa Cruz Biotechnology | WB, IP |
| 14-3-3σ Antibody | 18646 | IBL | WB, IP |
| mTOR(7C10) Rabbit mAb | 2983 | Cell Signaling | WB, IP |
| Raptor (24C12) Rabbit mAb | 2280 | Cell Signaling | WB, IP |
| Myc Antibody (9E10) | sc-40 | Santa Cruz Biotechnology | IP |
| Anti-GST (Mouse IgG2a-κ)　Monoclonal （GS019） | 04435-26 | NACALAI TESQUE | PD |
| Acetylated-Lysine Antibody | 9441 | Cell Signaling | WB, PD |
| Monoclonal Anti-phospho-HistoneH3(pSer28) antibody produced in Rat | H9908 | SIGMA | WB, IHC, IF, PD |
| Phospho-Histone H3(Ser10)(6G3) Mouse mAb Antibody | 9706 | CST | WB |
| Anti-Histone H3 (phospho S10) antibody | ab5176 | Abcam | IHC |
| AbFlex Histone H3, C-terminal antibody (rAb, Rabbit) | 91300 | ACTIVE MOTIF | WB |
| Axl(C89E7) Rabbit mAb | 8661 | Cell Signaling | WB |
| acetylated α Tubulin (6-11B-1) | sc-23950 | Santa Cruz | WB |
| αｰTubulin Antibody | T-9026 | SIGMA | WB |
| HDAC6 (D2E5) Rabbit mAb | 7558 | Cell Signaling | WB |
| Lamin A/C　Antibody | 2032 | Cell Signaling | WB |
| MEK 1/2 Antibody | 9122 | Cell Signaling | WB |
| Pan 14-3-3 Antibody | sc-629 | Santa Cruz Boitechnology | WB |
| Monoclonal Anti-Flag M2 antibody produced in mouse | F1804 | SIGMA | WB |
| E2F2 Antibody (TFE-25) | sc-9967 | Santa Cruz Boitechnology | IHC |
| EpCAM Antibody | ab71916 | Abcam | IHC |
| ID1 Antibody | ab203202 | Abcam | IHC |
| LIN28B Antibody | 4196 | Cell Signaling | WB |
| LIN28B Antibody | 16178-1-AP | Proteintech | IHC, IF |
| Cre recombinase antibody | MAB3120 | SIGMA （Millipore） | IHC |
| CD44v6 Antibody | AB2080 | SIGMA （Millipore） | IHC |
| Tri-Methyl-Histone H3 (Lys4)(C42D8) Rabbit mAb | 9751 | Cell Signaling | IHC |
| Purified Mouse Anti-E-Cadherin | 610181 | BD Biosciences | IHC |
| Ki67 Antibody | ab15580 | Abcam | IHC |

| **Supplementary Table 2: Primers** | |  |
| --- | --- | --- |
| **Gene Description** |  | **Sequence (5' -> 3')** |
| **E2F2** | Forward Primer | AGCTGGAACCGAGAGAACATG |
|  | Reverse Primer | ACACGACCAGGCGAAACC |
| **E2F8** | Forward Primer | AAGTACGCCGAGCAGATTATG |
|  | Reverse Primer | ATGTCTGGGTGTCCATTTGGG |
| **LIN28B** | Forward Primer | CATCTCCATGATAAACCGAGAGG |
|  | Reverse Primer | GTTACCCGTATTGACTCAAGGC |
| **ID1** | Forward Primer | ACACAAGATGCGATCGTCC |
|  | Reverse Primer | GGAATCCGAAGTTGGAACC |
| **SRSF1** | Forward Primer | CCGCAGGGAACAACGATTG |
|  | Reverse Primer | GCCGTATTTGTAGAACACGTCCT |
| **HPRT1** | Forward Primer | GCTTGCTGGTGAAAAGGACCTCTCGAAG |
|  | Reverse Primer | CCCTGAAGTACTCATTATAGTCAAGGGCAT |

# **Supplementary Figures
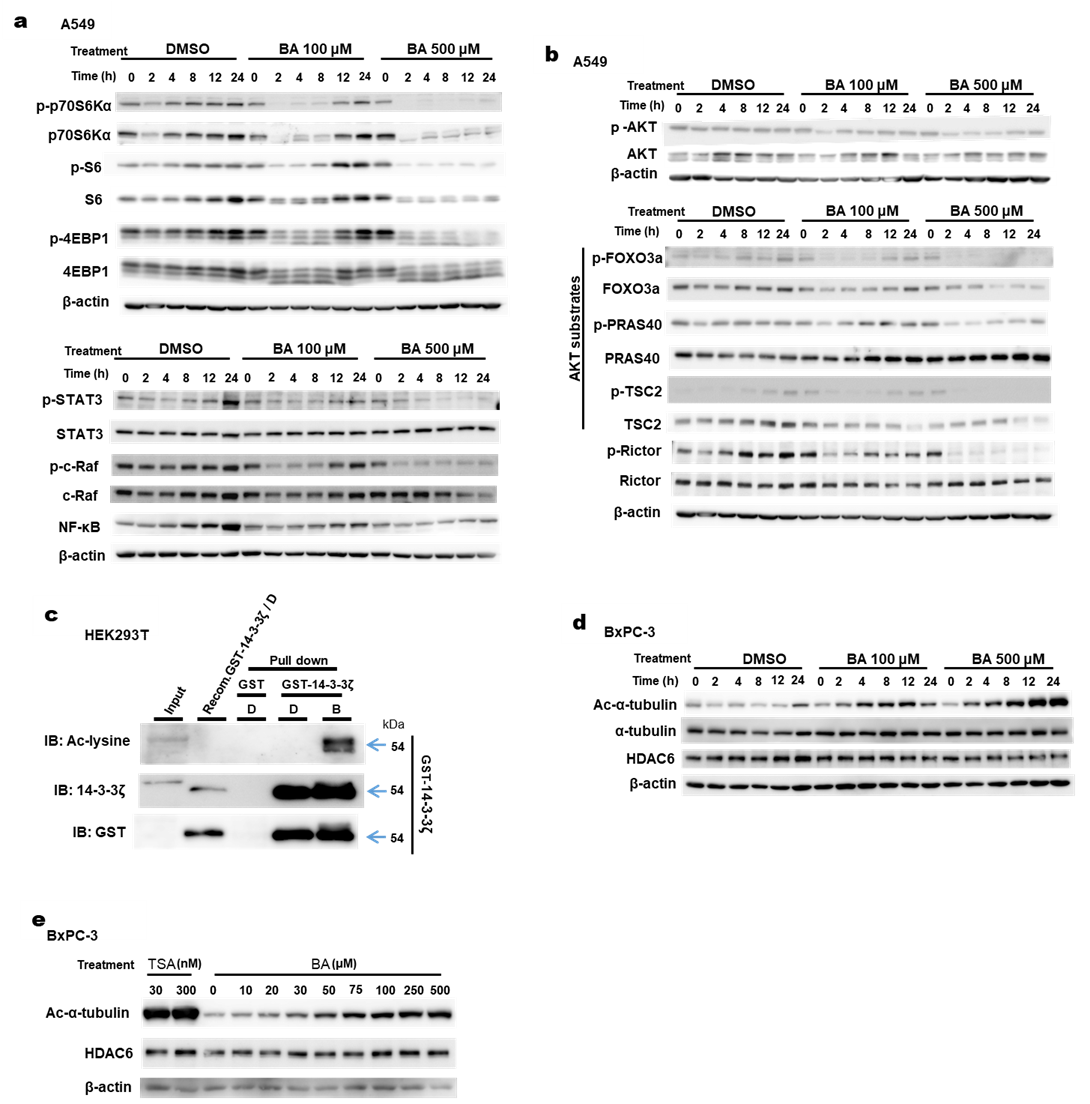
**

**Supplementary Figure 1, related to Fig. 2**

**a** Immunoblot analysis of total and phosphorylated (p-) forms of proteins involved in cancer-related signaling pathways in A549 cells treated with 100 or 500 µM BA or with DMSO vehicle for the indicated times. **b** Immunoblot analysis of total and phosphorylated forms of AKT, AKT substrates (FOXO3a, PRAS40, and TSC2), and Rictor in A549 cells treated as in **a**. **c** HEK293T cells treated with DMSO (D) or 1000 µM BA (B) for 100 min were lysed and subjected to a pull-down assay with GST or GST-tagged 14-3-3ζ. The resulting precipitates as well as the original cell lysates (Input) were subjected to immunoblot analysis with antibodies to acetyl-lysine, to 14-3-3ζ, and to GST. The lane labeled Recomb.GST–14-3-3ζ/D indicates recombinant GST-14-3-3 ζ without lysate with DMSO. **d** Immunoblot analysis of total and acetylated forms of α-tubulin as well as of HDAC6 in BxPC-3 cells treated as in a. **e** Immunoblot analysis of acetylated α-tubulin and HDAC6 in BxPC-3 cells treated with the indicated concentrations of the HDAC inhibitor tricostatin A (TSA) or BA for 7 h.

**
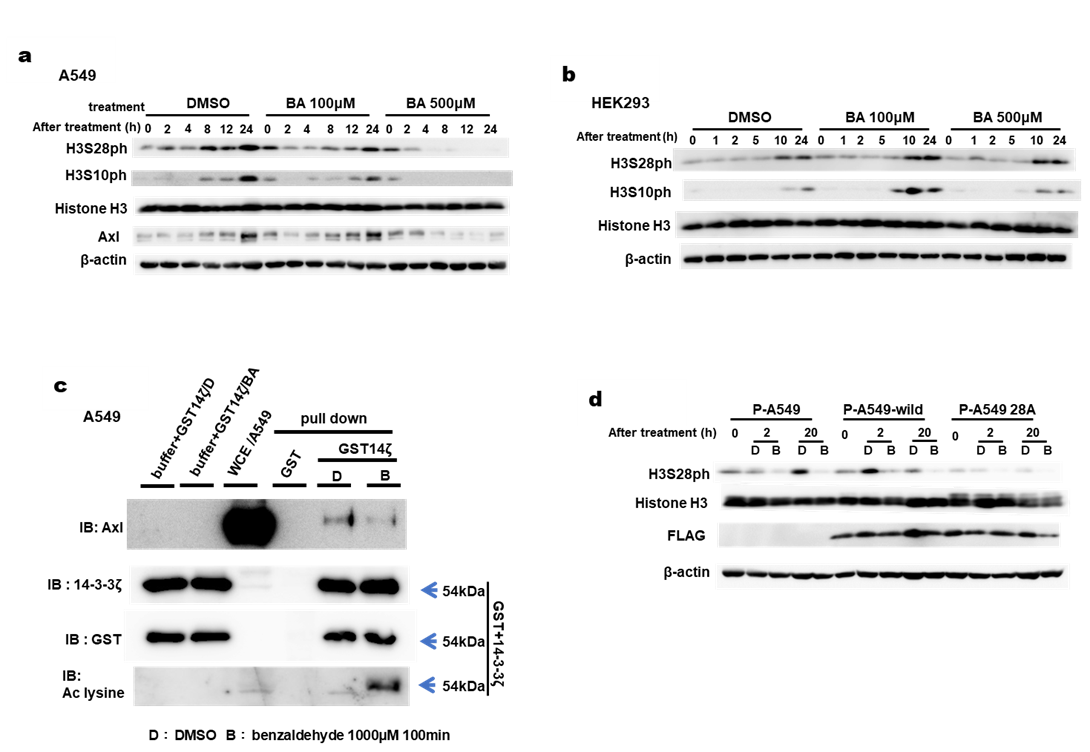
**

**Supplementary Figure 2, related to Figs. 2 and 3.**

**a**, **b** Immunoblot analysis of histone H3, H3S28ph, H3S10ph, and Axl in A549 (**a**) or HEK293 (**b**) cells treated with DMSO or 100 or 500 µM BA for the indicated times. **c** Pull-down assay with GST-tagged 14-3-3ζ (or GST) and high-salt extracts prepared from A549 cells that had been treated with DMSO (D) or 1000 µM BA (B) for 100 min. The pull-down material as well as the original cell lysates (Input) were subjected to immunoblot analysis with antibodies to Axl, 14-3-3ζ, GST, and acetyl-lysine. The lane labeled Recomb.GST–14-3-3ζ/D and Recomb.GST–14-3-3ζ/B indicate recombinant GST-14-3-3 ζ with DMSO, and recombinant GST-14-3-3 ζ with BA, respectively. **d** Immunoblot analysis of H3S28ph, histone H3, and FLAG in parental (P-) A549 cells expressing (or not) FLAG epitope–tagged WT or S28A mutant forms of H3 and treated with DMSO (D) or 500 µM BA (B) for the indicated times.

**
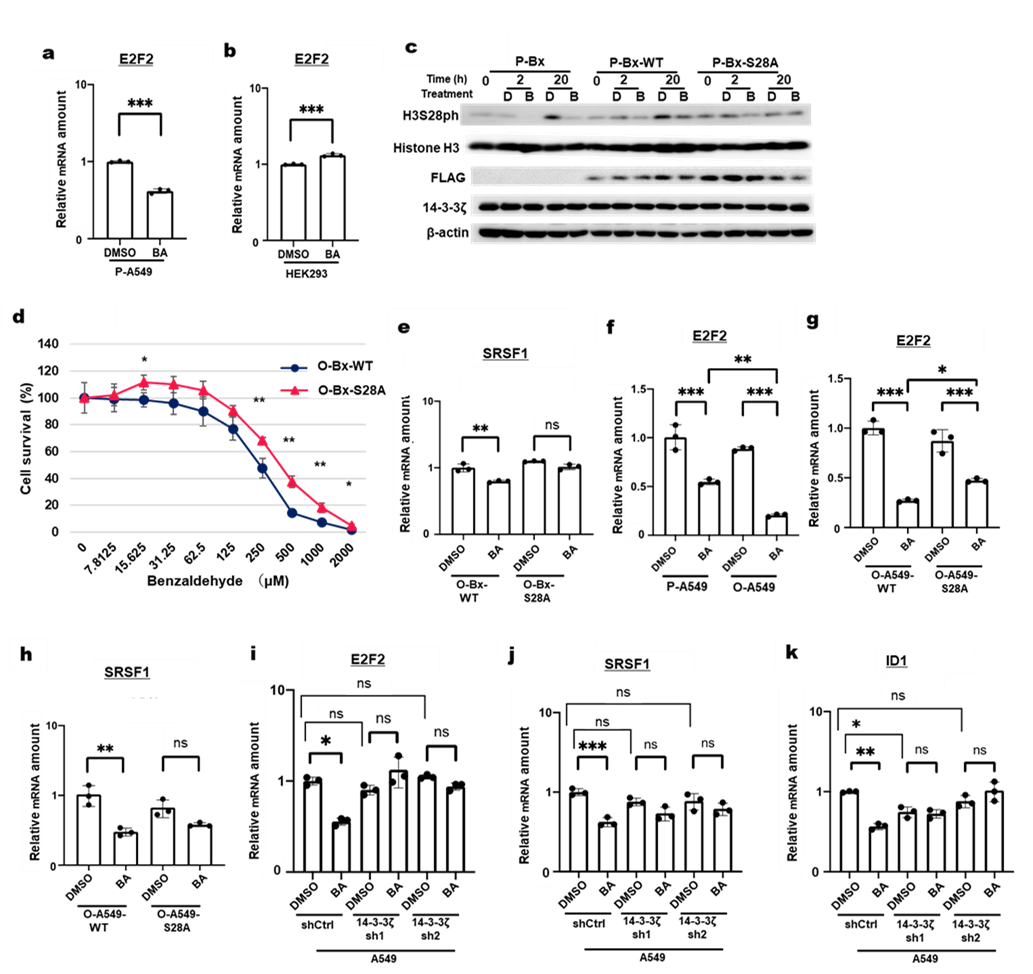
**

**Supplementary Figure 3, related to Fig. 4.**

**a**, **b** RT-qPCR analysis of *E2F2* mRNA in A549 (**a**) or HEK293 (**b**) cells treated with 500 µM BA or DMSO for 20 h. **c** Immunoblot analysis of parental (P-) BxPC-3 cells expressing (or not) FLAG-tagged WT or S28A mutant forms of histone H3 and incubated with 500 µM BA (B) or DMSO (D) for the indicated times. Cell lysates were probed with antibodies to histone H3, H3S28ph, FLAG, and 14-3-3ζ. **d** CellTiter-Glo assay for osimertinib-resistant (O-) BxPC-3 cells expressing WT or S28A mutant forms of H3 and incubated with the indicated concentrations of BA for 48 h. Data are means ± SD from n=3 independent experiments. *p < 0.05, **p < 0.01 versus the corresponding value for O-Bx-WT two-tailed Student’s t test). **e** RT-qPCR analysis of *SRSF1* mRNA in O-Bx-WT and O-Bx-S28A cells treated with 500 µM BA or DMSO for 20 h. **f** RT-qPCR analysis of *E2F2* mRNA in parental (P-) A549 and osimertinib-resistant (O-) A549 cells treated with 500 µM BA or DMSO for 20 h. **g**, **h** RT-qPCR analysis of *E2F2* (**g**) or *SRSF1* (**h**) mRNA in O-A549 cells expressing WT or S28A mutant forms of histone H3 and treated with 500 µM BA or DMSO for 20 h. **i**–**k** RT-qPCR analysis of *E2F2* (**i**), *SRSF1* (**j**), or *ID1* (**k**) mRNA in A549 cells expressing (or not) 14-3-3ζ shRNAs and treated with 500 µM BA or DMSO for 20 h. All RT-qPCR data are means ± SD from three independent experiments. *p < 0.05, **p < 0.01, ***p < 0.001; ns, not significant (two-tailed Student’s t test or one-way ANOVA followed by Tukey’s multiple-comparison test).

**
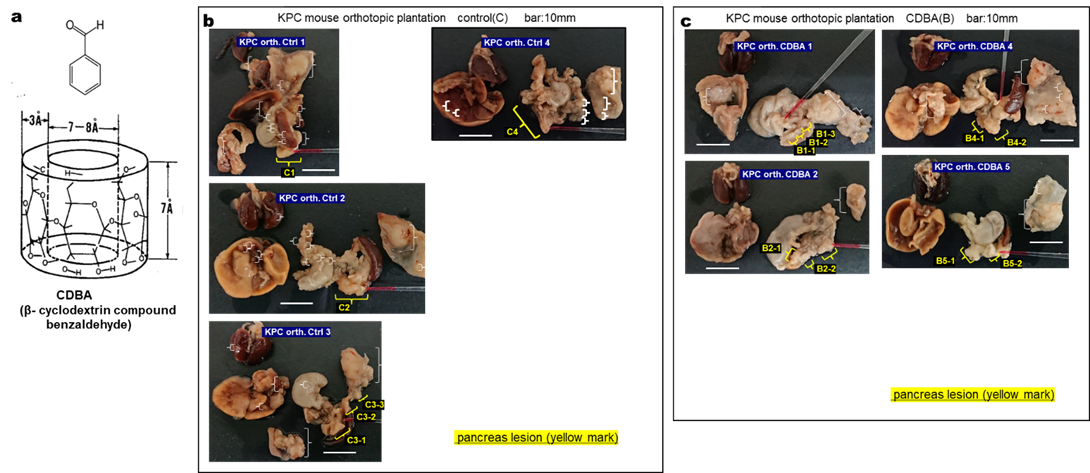
**

**Supplementary Figure 4, related to Fig. 5.**

**a** Chemical structure of the BA derivative CDBA. **b**, **c**, Anatomic findings for five and four pancreatic specimens of control (Ctrl) and CDBA-treated mice, respectively, of the KPC orthotopic transplantation model. Findings for the remaining mouse in each group are presented in Figure 5b. **d**, **e** Measurements of each pancreatic tumor (**d**) and total pancreatic tumor volume (**e**) for each mouse in the control group. **f**, **g** Measurements of each pancreatic tumor (**f**) and total pancreatic tumor volume (**g**) for each mouse in the CDBA group.

**
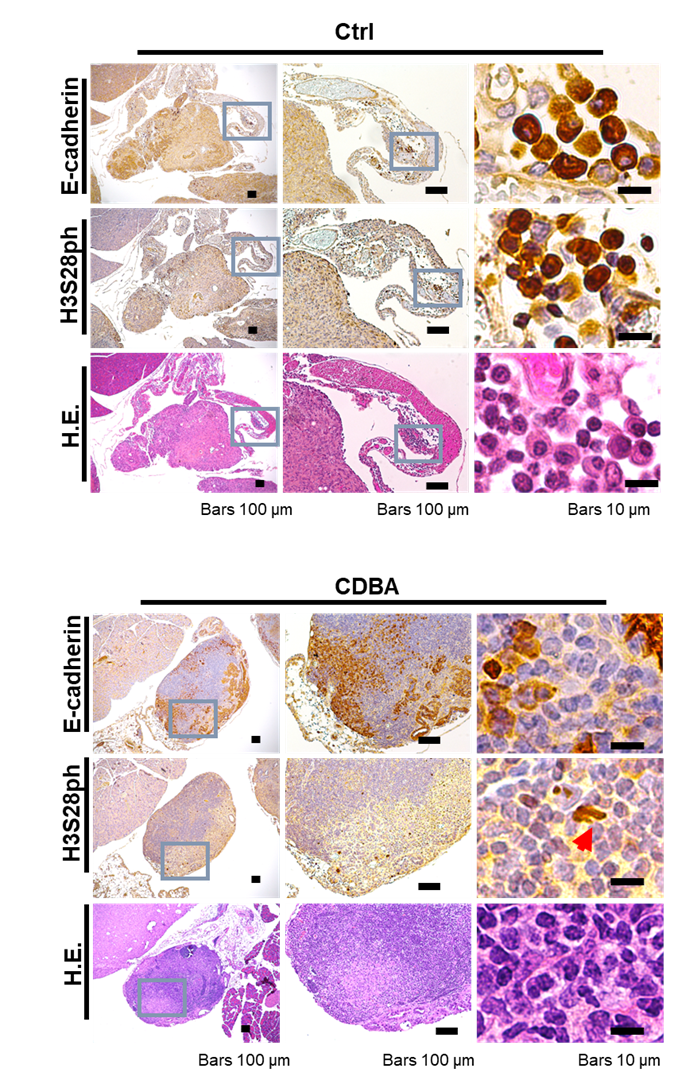
**

**Supplementary Figure 5, related to Fig. 6.**

Immunohistochemical analysis of E-cadherin and H3S28ph as well as H.E. staining for serial sections of pancreatic tumor tissue from the mouse KPC orthotopic transplantation model. The tissue was derived from control (Ctrl) mice and mice treated with CDBA. The red arrow indicates mitotic catastrophe associated with damaged chromatin.

**
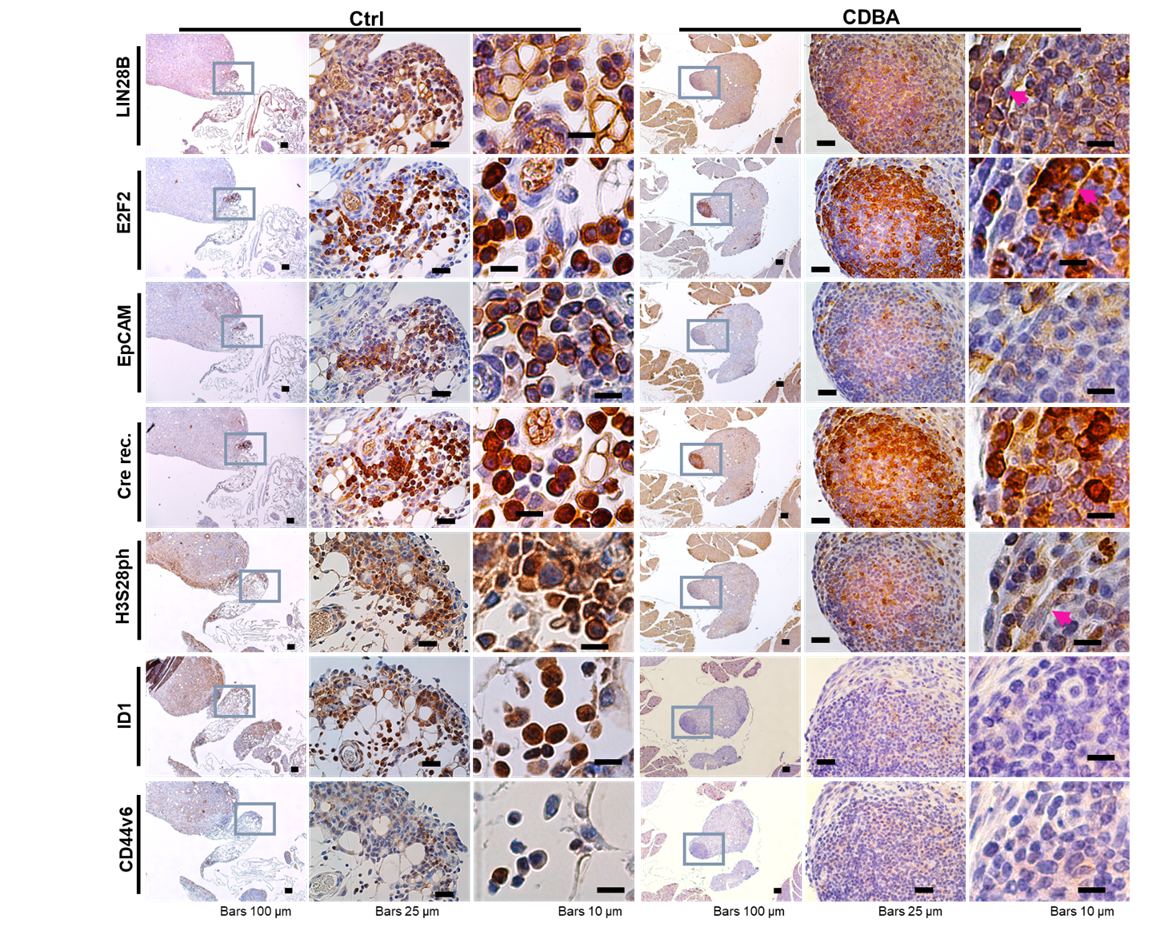
**

**Supplementary Figure 6, related to Figs. 6 and 7.**

Immunohistochemical analysis of LIN28B, E2F2, EpCAM, Cre recombinase, H3S28ph, ID1, and CD44v6 in serial sections of pancreatic tumor tissue from the mouse KPC orthotopic transplantation model. The tissue was derived from control (Ctrl) and CDBA-treated mice. Pink arrows indicate pearl formation by epithelial-like cells.

**
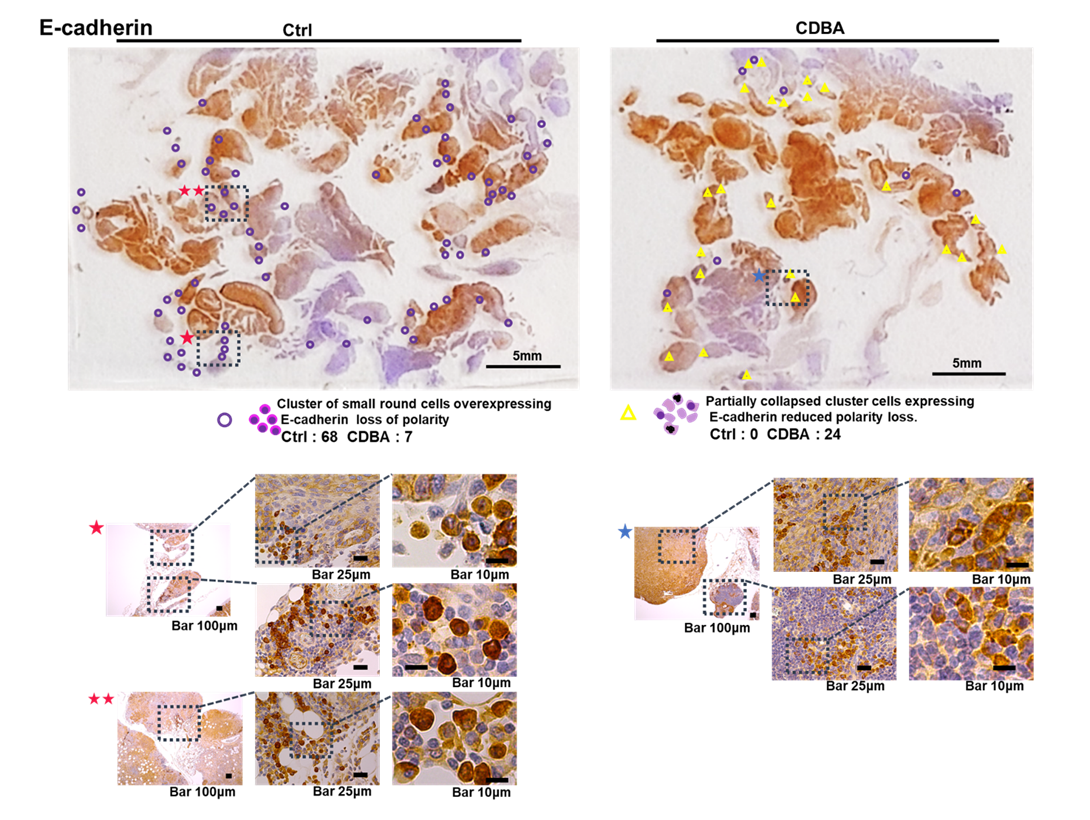
**

**Supplementary Figure 7, related to Figs. 6 and 7.**

Immunohistochemical analysis of pancreatic tumor tissues from the mouse KPC orthotopic transplantation model. Images show the full field of view of tissue slides derived from control (Ctrl) mice (n=6) and mice treated with CDBA (n=5).

**E-cadherin :** Marked with purple circles are clusters of small, round cells overexpressing E-cadherin with loss of polarity, not showing the canonical expression on the cell membrane. Marked with yellow triangles are partially collapsed cell clusters expressing E-cadherin with reduced polarity loss.

**
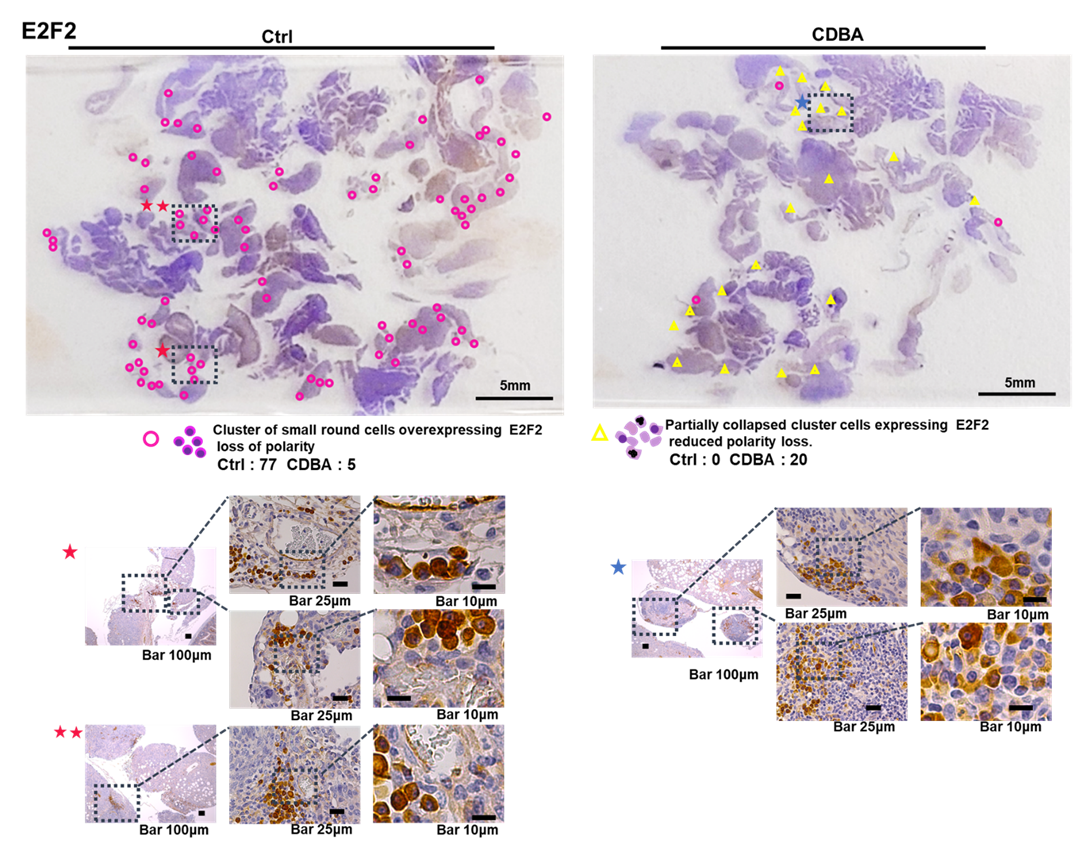
**

**Supplementary Figure 8, related to Figs. 6 and 7.**

Immunohistochemical analysis of pancreatic tumor tissues from the mouse KPC orthotopic transplantation model. Images show the full field of view of tissue slides derived from control (Ctrl) mice (n=6) and mice treated with CDBA (n=5).

**E2F2 :** Marked with pink circles are clusters of small, round cells overexpressing E2F2 with loss of polarity. Marked with yellow triangles are partially collapsed cell clusters expressing E2F2 with reduced polarity loss.

**
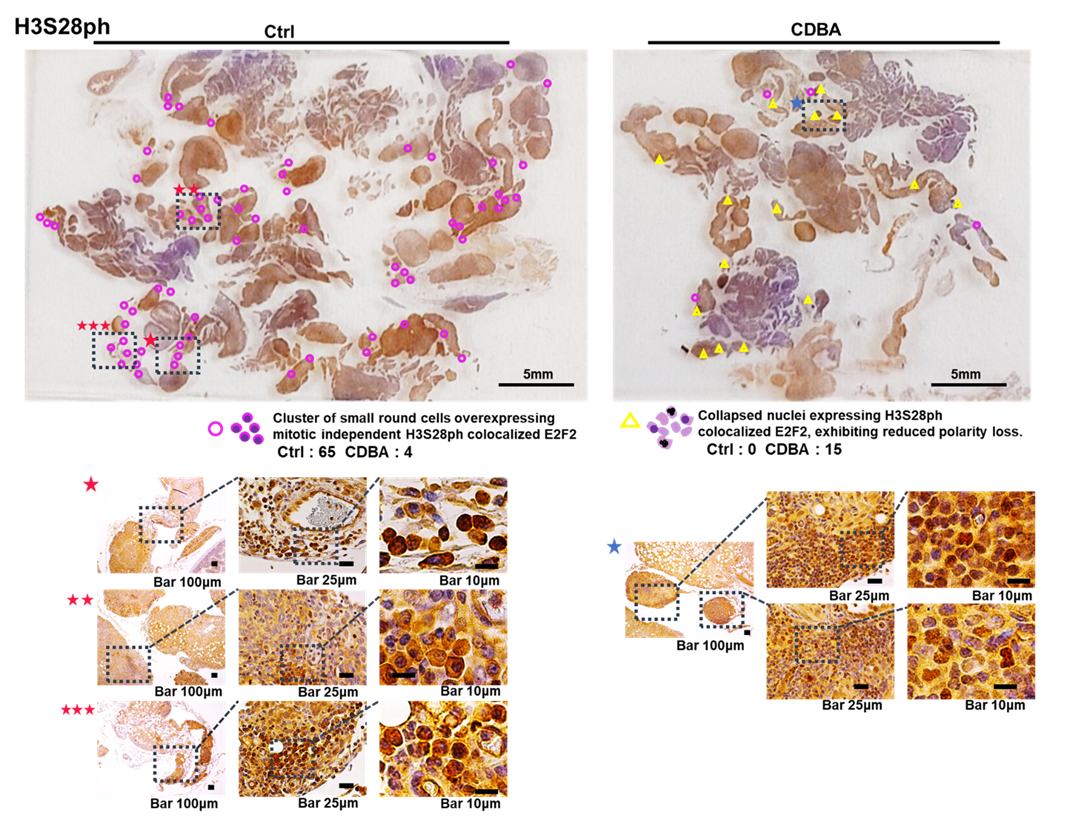
**

**Supplementary Figure 9, related to Figs. 6 and 7.**

Immunohistochemical analysis of pancreatic tumor tissues from the mouse KPC orthotopic transplantation model. Images show the full field of view of tissue slides derived from control (Ctrl) mice (n=6) and mice treated with CDBA (n=5).

**H3S28ph :** Marked with purple circles are clusters of small, round cells overexpressing mitosis-independent H3S28ph colocalized with E2F2 and showing loss of polarity. Marked with yellow triangles are partially collapsed nuclei expressing H3S28ph colocalized with E2F2, exhibiting reduced polarity loss.

**
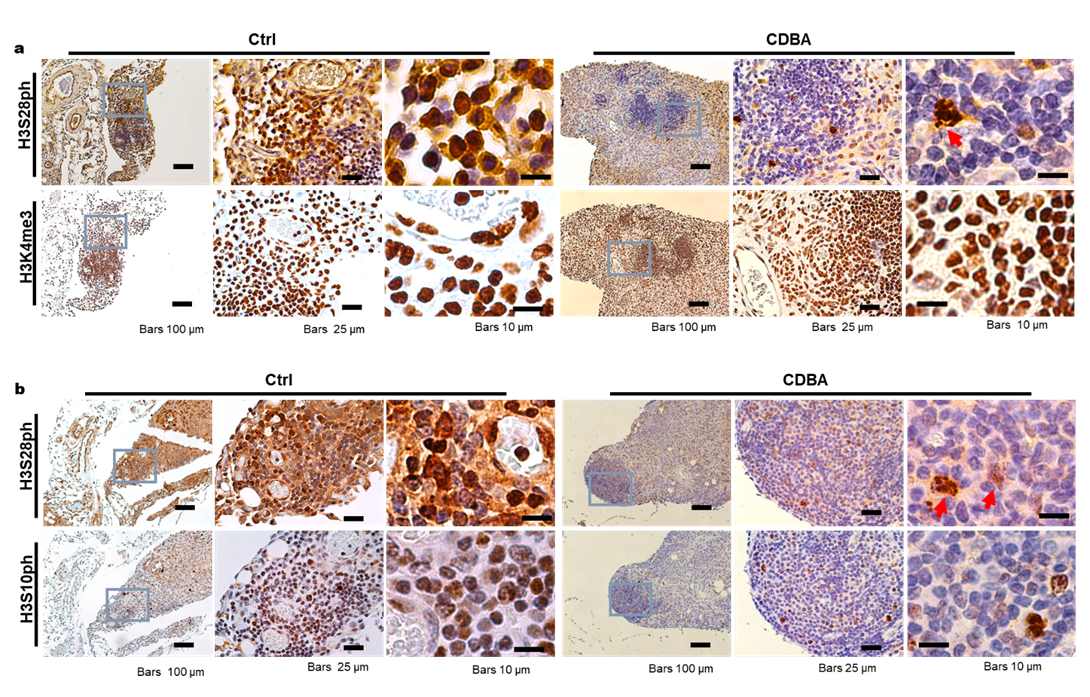
**

**Supplementary Figure 10, related to Figs. 6 and 7.**

Immunohistochemical analysis of H3S28ph and H3K4me3 (**a**) and of H3S28ph and H3S10ph (**b**) in serial sections of pancreatic tumor tissue from the mouse KPC orthotopic transplantation model. The tissue was derived from control (Ctrl) and CDBA-treated mice, Red arrows indicate mitotic catastrophe associated with damaged chromatin.

**
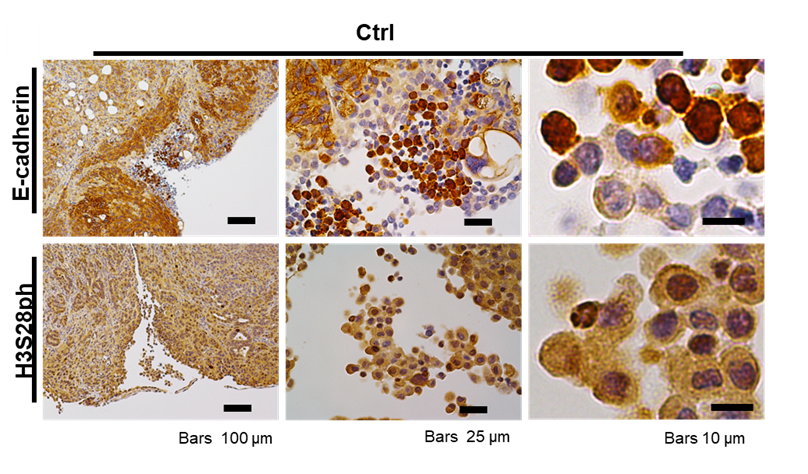
**

**Supplementary Figure 11, related to Figs. 6 and 7.**

Immunohistochemical analysis of E-cadherin and H3S28ph in peritoneal disseminated tissue from control (Ctrl) mice of the KPC orthotopic transplantation model. Rounded cells in the disseminated state manifest EMP-like features.

**
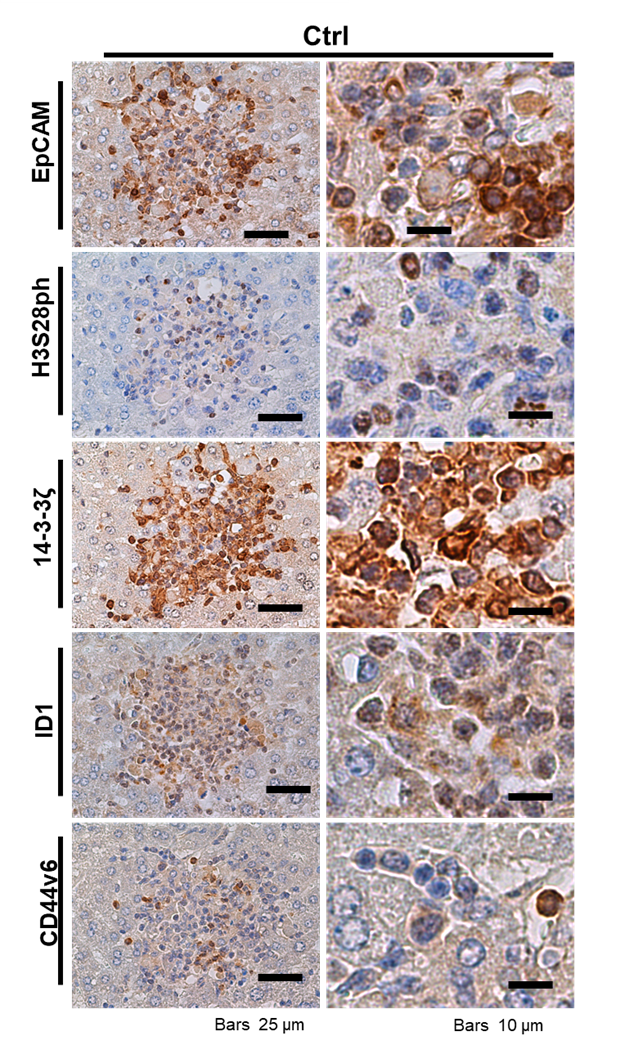
**

**Supplementary Figure 12, related to Figs. 5 to 7.**

Immunohistochemical analysis of EpCAM, H3S28ph, 14-3-3ζ, ID1, and CD44v6 in serial sections of liver tissue from control (Ctrl) mice of the KPC orthotopic transplantation model. Small accumulations of cells positive for EpCAM, H3S28ph, 14-3-3ζ, and ID1 were apparent in the liver, suggestive of metastasis.
